# Review of the risk of cancer following low and moderate doses of sparsely ionising radiation received in early life in groups with individually estimated doses

**DOI:** 10.1016/j.envint.2021.106983

**Published:** 2021-12-24

**Authors:** Mark P. Little, Richard Wakeford, Simon D. Bouffler, Kossi Abalo, Michael Hauptmann, Nobuyuki Hamada, Gerald M. Kendall

**Affiliations:** a Radiation Epidemiology Branch, National Cancer Institute, Bethesda, MD 20892-9778, USA; b Centre for Occupational and Environmental Health, Faculty of Biology, Medicine and Health, The University of Manchester, Ellen Wilkinson Building, Oxford Road, Manchester M13 9PL, UK; c Radiation Effects Department, UK Health Security Agency (UKHSA), Chilton, Didcot OX11 0RQ, UK; d Laboratoire d’Épidémiologie, Institut de Radioprotection et de Sûreté Nucléaire, BP 17, 92262 Fontenay-aux-Roses Cedex, France; e Institute of Biostatistics and Registry Research, Brandenburg Medical School Theodor Fontane, Fehrbelliner Strasse 38, 16816 Neuruppin, Germany; f Radiation Safety Unit, Biology and Environmental Chemistry Division, Sustainable System Research Laboratory, Central Research Institute of Electric Power Industry (CRIEPI), 2-11-1 Iwado-kita, Komae, Tokyo 201-8511, Japan; g Cancer Epidemiology Unit, Oxford Population Health, University of Oxford, Richard Doll Building, Old Road Campus, Headington, Oxford, OX3 7LF, UK

**Keywords:** Radiation, Childhood, *In utero*, Cancer risk, Radiobiology

## Abstract

**Background:**

The detrimental health effects associated with the receipt of moderate (0.1–1 Gy) and high (>1 Gy) acute doses of sparsely ionising radiation are well established from human epidemiological studies. There is accumulating direct evidence of excess risk of cancer in a number of populations exposed at lower acute doses or doses received over a protracted period. There is evidence that relative risks are generally higher after radiation exposures *in utero* or in childhood.

**Methods and findings:**

We reviewed and summarised evidence from 60 studies of cancer or benign neoplasms following low- or moderate-level exposure *in utero* or in childhood from medical and environmental sources. In most of the populations studied the exposure was predominantly to sparsely ionising radiation, such as X-rays and gamma-rays. There were significant (*p* < 0.001) excess risks for all cancers, and particularly large excess relative risks were observed for brain/CNS tumours, thyroid cancer (including nodules) and leukaemia.

**Conclusions:**

Overall, the totality of this large body of data relating to *in utero* and childhood exposure provides support for the existence of excess cancer and benign neoplasm risk associated with radiation doses < 0.1 Gy, and for certain groups exposed to natural background radiation, to fallout and medical X-rays *in utero*, at about 0.02 Gy.

## 1. Introduction

Although moderate and high doses of sparsely ionising radiation (such as X-rays and gamma-rays), when received at a high dose-rate, are known to be associated with elevated cancer risks (Armstrong et al. 2012; Committee to Assess Health Risks from Exposure to Low Levels of Ionizing Radiation 2006; International Commission on Radiological Protection (ICRP) 2007; United Nations Scientific Committee on the Effects of Atomic Radiation (UNSCEAR) 2008), less is known about any risks arising from exposures at lower doses and dose rates.

There is growing evidence in the Japanese atomic bomb survivors (Grant et al. 2017; Little et al. 2020) and in groups receiving medical diagnostic exposures or radiation therapy of an excess risk of cancer following lower levels of exposure to radiation, particularly among those exposed in childhood (Little et al. 2018b; Lubin et al. 2017). The pioneering case-control study of Stewart *et al* (Bithell and Stewart 1975; Stewart et al. 1956; Stewart et al. 1958), which became known as the Oxford Survey of Childhood Cancers (OSCC), suggested that there might be excess risk of most types of childhood cancer associated with antenatal exposure to doses of about 0.01–0.03 Gy of X-rays; however, the interpretation of the association found by this and similar case-control studies has been controversial, with potential for recall and selection biases and for confounding (Brent 2014; International Commission on Radiological Protection (ICRP) 2003; United Nations Scientific Committee on the Effects of Atomic Radiation (UNSCEAR) 2008). More recently there have been a number of studies of childhood cancer associated with natural background exposure to gamma radiation (with cumulative doses generally in the range of at most a few tens of mGy), some (but not all) of which have observed excess cancer risks (Kendall et al. 2021; Mazzei-Abba et al. 2020).

By far the largest part (>80%) of man-made radiation exposure (apart from patients receiving radiotherapy) to children in the US is from computed tomography (CT) scan use, comprising a collective effective dose of about 18,000 person Sv in 2016, with much smaller contributions from conventional radiography (∼1300 person Sv), fluoroscopy (∼700 person Sv), nuclear imaging (∼700 person Sv), and image-guided interventions (∼300 person Sv) (National Council on Radiation Protection and Measurements (NCRP) 2019). Both in the US and Canada rates of CT scan use in children have stabilised since the early 2000s, with some signs of reduction since about 2006 (Smith-Bindman et al. 2019), in contrast to trends in the adult population in the US and elsewhere where rates have carried on increasing (National Council on Radiation Protection and Measurements (NCRP) 2009; 2019; Smith-Bindman et al. 2019). There have been a number of studies evaluating risks of cancer after CT scanning in childhood, many of which have indicated some excess risk (Berrington de Gonzalez et al. 2016; Journy et al. 2015; Journy et al. 2016; Kojimahara et al. 2020; Krille et al. 2015; Mathews et al. 2013; Meulepas et al. 2019; Pearce et al. 2012) although the interpretation of these findings is not straightforward (Boice 2015; Walsh et al. 2014).

There have been a number of recent reviews of this low and moderate dose literature, in particular by the National Council on Radiation Protection and Measurements (NCRP) (National Council on Radiation Protection and Measurements (NCRP) 2018; Shore et al. 2018; Shore et al. 2019) and by a large group of collaborators coordinated by the National Cancer Institute (NCI) (Berrington de Gonzalez et al. 2020; Daniels et al. 2020; Gilbert et al. 2020; Hauptmann et al. 2020; Linet et al. 2020; Schubauer-Berigan et al. 2020), although most studies surveyed in both cases related to exposure in adulthood.

Childhood cancers have never been common diseases and recent decades have seen great improvements in therapy (Stiller 2007). Nevertheless, they remain diseases of concern, particularly due to the temporal and spatial variations in incidence (“clusters”) that have been observed in, for example, childhood leukaemia (Steinmaus et al. 2004). In this paper we shall review cancer risks following exposure to sparsely ionising (low linear energy transfer (LET)) radiation exposure early in life (*in utero* and in childhood) in many of these low and moderate dose studies, and by a meta-analysis quantitatively assess the degree of compatibility of relative risk estimates derived from the main studies. As such, the focus of this review is quite distinct from the recent reviews of low dose risk by the NCRP (National Council on Radiation Protection and Measurements (NCRP) 2018) and by the NCI (Berrington de Gonzalez et al. 2020; Daniels et al. 2020; Gilbert et al. 2020; Hauptmann et al. 2020; Linet et al. 2020; Schubauer-Berigan et al. 2020), which concentrated on all-age exposure with the former conducted in the context of radiological protection, and specifically did not assess *in utero* exposure in any depth; however, the issue of risks after radiation exposure in childhood has been dealt with in a number of other reviews (Linet et al. 2009; United Nations Scientific Committee on the Effects of Atomic Radiation (UNSCEAR) 2013). In the Discussion we shall also briefly review the radiobiology to assess the biological plausibility of these epidemiological associations.

## 2. Methods

### 2.1. Literature review

A literature search of PubMed was last performed on 16th May 2021 using the search terms given in the Supplementary Methods. Additionally, recent UNSCEAR reports (United Nations Scientific Committee on the Effects of Atomic Radiation (UNSCEAR) 2008; 2013; 2018) were scanned to assess additional literature, as well as recent review articles (Kendall et al. 2021; Linet et al. 2009; Linet et al. 2012; Wakeford and Bithell 2021). We restricted attention to those studies of persons exposed *in utero* or in childhood (age 20 y or less) and with individually estimated organ/tissue doses. A further restriction was either that maximum cumulative doses (or if this could not be determined, mean cumulative doses) should not exceed the conventional definitions of low doses, <0.1 Gy, or moderate doses, 0.1–1 Gy (Harrison et al. 2021; Little et al. 2021a; United Nations Scientific Committee on the Effects of Atomic Radiation (UNSCEAR) 2015), or that the maximum dose rate should not exceed 0.005 Gy per hour (the conventional upper limit for low dose rate (United Nations Scientific Committee on the Effects of Atomic Radiation (UNSCEAR) 2018) or 0.1 Gy per hour (which we take as the upper limit for moderate dose rate). Justification for these limits will be found in the Supplementary Methods.

### 2.2. Meta-analysis

Meta-analysis was conducted of the studies, of *in utero* and postnatal exposures, outlined in Tables 1–5; the basis of all estimations of radiation risk in this latter analysis is the value of excess relative risk (ERR) per unit of absorbed dose of radiation exposure (ERR per Gy). For absorbed dose, most publications employed unweighted radiation dose (Gy), but some use weighted dose, for example in the LSS to account for the higher biological effectiveness of neutrons compared with photons (Shimizu et al. 2010; Grant et al. 2017). Wherever possible the OR, RR or ERR were taken directly from the relevant publication; further details are given in Tables 1–5 and Supplementary Tables S1-S2. There are grounds for thinking that risks of thyroid nodule and thyroid cancer are not dissimilar, as for example suggested by the Ukraine *in utero* data of Hatch *et al* (Hatch et al. 2019), likewise among postnatally exposed groups in Belarus (Cahoon et al. 2017a; Zablotska et al. 2011). For this reason we group thyroid cancer and thyroid nodules together in all meta-analyses. Further details of data exclusions and of how the data abstraction was performed for particular studies are given in the Supplementary Methods.

An aggregate estimate of ERR per Gy was computed across subsets of these studies using random effects models and standard statistical methods. Random effects models were fitted by restricted maximum likelihood (REML) because of the theoretically superior performance, in particular, the absence of bias in the estimate of variance (Bartlett and Fowler 1937; Viechtbauer 2005). Results are given in Table 6 and Supplementary Tables S3, S4. However, for certain analyses (Table 7, Supplementary Tables S5) maximum-likelihood fits were used, as these facilitate comparison of nested models (in particular, to test against improvement over the null). Values of ERR per Gy derived from the meta-analysis are given in Tables 6, 7 and Supplementary Tables S3-S5 for major cancer subtypes (leukaemia, lymphoma, brain/central nervous system (CNS), etc.), by *in utero* vs postnatal exposure and by level of maximum dose or maximum dose rate, into low (L), medium (M), or high (H), using the classification by dose (L/M/H) and dose rate (L/M/H) in columns 3 and 5, respectively, of Tables 1–5. We undertook sensitivity analyses in which we refitted:

- thyroid nodule data < 0.799 Gy of Hatch *et al* (Hatch et al. 2019);
- thyroid cancer data < 0.284 Gy of Kopecky *et al* (Kopecky et al. 2006);
- Cardis *et al* (Cardis et al. 2005) thyroid cancer data using a linear model restricted to < 1 Gy;
- Lubin *et al* (Lubin et al. 2017) data restricted to < 0.1 Gy;
- Preston *et al* (Preston et al. 2007) brain/CNS and breast cancer data restricted to < 0.1 Gy;
- Cahoon *et al* (Cahoon et al. 2017b) lung cancer data restricted to < 0.1 Gy.

These we term the “lower dose risks”. These we contrasted with using instead:

- the full dose range thyroid nodule data of Hatch *et al* (Hatch et al. 2019);
- the full dose range thyroid cancer data of Kopecky *et al* (Kopecky et al. 2006);
- Cardis *et al* (Cardis et al. 2005) thyroid cancer data using a linear model restricted to < 2 Gy;
- Lubin *et al* (Lubin et al. 2017) data restricted to < 0.2 Gy;
- Preston *et al* (Preston et al. 2007) brain/CNS and breast cancer data restricted to < 1 Gy; and
- Cahoon *et al* (Cahoon et al. 2017b) lung cancer data restricted to < 1 Gy.

These we term the “higher dose risks”.

All statistical models were fitted using the metafor package (Viechtbauer 2010; 2020) in R (R Project version 3.6.1 2019). Further details of the statistical methods are given in the Supplementary Methods.

## 3. Results

The second stage of the literature review yielded 49 studies in which radiation exposure has been quantitatively assessed, whether *in utero* or in childhood (Tables 1–5). Supplementary Table S6 gives details of a further 11 studies of natural background radiation that are not included in Table 1, generally because they were not informative or because their results were effectively subsumed within larger studies; also given in Supplementary Table S6, for completeness, are details of those 6 studies that are also included in Table 1.

### 3.1. Risks of in utero exposure

There are strong estimates of excess risk of cancer in childhood in the OSCC study of Bithell and Stiller (Bithell and Stiller 1988) and of Bithell (Bithell 1993) (Table 2, Supplementary Table S7), at doses that likely do not exceed 0.03 Gy, and borderline significant indications of excess risk for lympho-haematopoietic malignancies in the range of attained ages up to 61 years in the Southern Urals study of Schüz *et al* (Schüz et al. 2017) and for all thyroid nodules (mainly benign) at an attained age of 25–30 years in the Ukraine ^131^I-exposed cohort of Hatch *et al* (Hatch et al. 2019) (Table 3, Supplementary Table S7). There are weaker indications of excess brain tumour risk in a case-control study of medical diagnostic exposures (Pasqual et al. 2020) (Table 2, Supplementary Table S7), for solid cancer in the *in utero* exposed Japanese atomic bomb survivors in the incidence study of Preston *et al* (Preston et al. 2008) at attained age of 12–55 years and in the mortality study of Sugiyama *et al* (Sugiyama et al. 2021) at attained age of 5–67 years (Table 4, Supplementary Table S7), and for lymphoma, leukaemia and solid cancer in the offspring of US radiologic technologists (Johnson et al. 2008) at attained age up to 20 years (Table 3, Supplementary Table S7).

### 3.2. Risks of radiation exposure in childhood

#### 3.2.1. Risks associated with environmental radiation exposure

##### 3.2.1.1. Naturally occurring environmental exposures.

A large number of studies of natural background radiation and childhood cancer have been conducted, as shown in Table 1 and Supplementary Table S6. The maximum doses are generally very low, in no case exceeding 0.05 Gy (Table 1). Among the main studies listed in Table 1, all are of natural background gamma radiation, but some also include assessment of the risks of radon exposure (Berlivet et al. 2021; Berlivet et al. 2020; Demoury et al. 2017; Kendall et al. 2013). All these studies, and two studies that assessed only gamma radiation (Nikkilä et al. 2016; Spycher et al. 2015) are of European national populations, and are register based. Most studies do not yield significant excess risks, the only exceptions being the British study of Kendall *et al* (Kendall et al. 2013) and the Swiss study of Spycher *et al* (Spycher et al. 2015). We note that since the original database search was conducted an updated Swiss study has been published (Mazzei-Abba et al. 2021), which reported very similar relative risk estimates to those of Spycher *et al* (Spycher et al. 2015) and we judge that the meta-analysis would be little affected. The small Finnish study of Nikkilä *et al* (Nikkiläet al. 2016) is probably of limited statistical power, but the French studies are of much larger populations (Berlivet et al. 2021; Berlivet et al. 2020; Demoury et al. 2017). The small size of most of the studies in Supplementary Table S6 means that they have little realistic chance of detecting an effect of radiation exposure.

##### 3.2.1.2. Exposures due to man-made environmental contamination.

The Chernobyl nuclear accident in northern Ukraine in 1986 resulted in large releases of radioisotopes of iodine and caesium, and other radionuclides to a lesser extent, resulting in a mixture of internal and external exposure, particularly to parts of the populations of Ukraine, Belarus and Russia (United Nations Scientific Committee on the Effects of Atomic Radiation (UNSCEAR) 2011). One of the main health consequences has been the markedly elevated incidence of thyroid cancer, largely due to intakes of radioiodine by children (Brenner et al. 2011). However, as noted in the Methods, the thyroid doses in most of these studies include both high doses and high dose rates; an exception in this respect is the Belarus and Russia thyroid cancer case-control study of Cardis *et al* (Cardis et al. 2005), which did consider risks over lower dose (and dose rate) ranges (Table 5).

More relevant to the present review are two case-control studies of childhood leukaemia among those exposed *in utero* or under the age of 6 years while living in heavily contaminated areas of the former USSR at the time of the Chernobyl accident; the mean dose to the active bone marrow (ABM) was around 10 mGy (Davis et al. 2006; Noshchenko et al. 2010). There are modest excess risks in the Russian and Belarusian components of the study of Davis *et al* (Davis et al. 2006) and the Ukrainian study of Noshchenko *et al* (Noshchenko et al. 2010), only the second of these significant (Table 5). Raised, albeit non-significant, risks were observed in a study of childhood leukaemia in Utah and fallout from the Nevada Test Site (Stevens et al. 1990) (Table 5). A cohort study in Utah, Nevada and Arizona of thyroid disease and childhood exposure to fallout from the Nevada Test Site found a positive association for thyroid neoplasms (Lyon et al. 2006) (Table 5). By contrast, a cohort study of persons exposed in early life to ^131^I releases from the Hanford nuclear site (Davis et al. 2004) yielded virtually no excess risks of thyroid cancer or benign thyroid nodules (Table 5), but this study probably had only limited power to detect the increased risks predicted by studies of external exposure in childhood (Table 4).

#### 3.2.2. Risks associated with medical diagnostic exposure and other low and moderate dose radiation exposure

There have been a number of studies evaluating risks of cancer after diagnostic CT scan exposure in childhood, some of which have yielded excess risks of various cancers (Berrington de Gonzalez et al. 2016; Journy et al. 2015; Journy et al. 2016; Kojimahara et al. 2020; Krille et al. 2015; Mathews et al. 2013; Meulepas et al. 2019; Nikkilä et al. 2018; Pearce et al. 2012), and summarised in Table 2. Most studies only assessed risk of leukaemia and brain tumours (Table 2). Only in the Finnish case-control study (Nikkilä et al. 2018), the Australian study (Mathews et al. 2013) and the UK study (Pearce et al. 2012), the low dose part of which is subsumed in the study of Little *et al* (Little et al. 2018b), was the excess risk of leukaemia statistically significant. In the UK study (Berrington de Gonzalez et al. 2016; Pearce et al. 2012), the Australian study (Mathews et al. 2013), the German study (Krille et al. 2015) and the Dutch study (Meulepas et al. 2019) there were significant excess risks of brain tumours (Table 2).

Table 2 shows leukaemia risks in the < 0.1 Gy pooled dataset of Little *et al* (Little et al. 2018b), also thyroid cancer incidence following external exposure in childhood at thyroid doses of < 0.2 Gy and < 0.1 Gy (Lubin et al. 2017). Risks in both studies are statistically significantly elevated (Table 2). Table 4 shows the paediatrically exposed moderate dose range brain and breast cancer data of Preston *et al* (Preston et al. 2007), the lung cancer data of Cahoon *et al* (Cahoon et al. 2017b) and the solid cancer data of Grant *et al* (Grant et al. 2017), all from the LSS cohort. The risks in these datasets are generally compatible with those in the medical diagnostic studies, although the central estimates of risk tend to be higher in the LSS (Tables 2, 4).

### 3.3. Meta-analysis of cancer risks associated with radiation exposure in early life

#### 3.3.1. Restricted maximum likelihood (REML) and maximum likelihood analyses

The REML analysis of Table 6 in relation to postnatal exposure suggests that, overall, there were significantly elevated risks of all cancers and brain/CNS tumours (*p* < 0.05), with marginally significant (*p* = 0.108) elevation of risk for leukaemia. Risks for lymphoma and the remainder category (cancers other than leukaemia, lymphoma, brain/CNS tumours) were markedly lower, and were not statistically significant (*p* > 0.5). In relation to *in utero* exposure there was (at least using the lower dose analysis) significant excess risk for thyroid cancer and thyroid nodules (*p* < 0.05), and large (but non-significant (*p* > 0.2)) excess risks for brain/CNS tumours. [Note: the meaning of “higher dose” studies and “lower dose” studies is defined in the Methods.] For most postnatal exposure endpoints there was significant inter-study heterogeneity, as indicated by the *Q* statistic, although that was not the case in relation to *in utero* exposure (Table 6). The *I*^2^ statistic was often substantial for many postnatal exposure endpoints, a number above 40%, implying that a material proportion of the variance was due to inter-study heterogeneity; however, this was generally not the case for *in utero* exposure. Analysis of the data for *in utero* exposed subjects using adjustments to the ERR for attained age yielded much larger risks (Supplementary Table S4) than those using the unadjusted data (Table 6), but only for thyroid nodules was there a significant positive trend when using the lower dose estimates (*p* = 0.033).

Further maximum likelihood analyses were performed in order to assess the significance of certain contrasts between lower and higher dose meta-analyses, exhibited in Table 7. These suggest that for thyroid cancer (including thyroid nodules) there was significantly lower risk (*p* = 0.033) associated with postnatal exposure compared with *in utero* exposure when using the lower dose estimates. Lung cancer and thyroid (including nodules) risk were significantly higher at lower levels of dose (*p* = 0.031, *p* = 0.001 respectively) using the lower dose set of risk estimates. Lung cancer risk was significantly higher for low dose-rate exposures than for moderate and high dose-rate exposures (*p* = 0.031), although information was only available for the lower dose set of risk estimates, whereas for thyroid (including nodules) risks were significantly higher (*p* < 0.001) for the moderate and high dose-rate exposures than for lower dose-rate exposure. However, the problems of convergence with all the thyroid cancer/thyroid nodules model fits complicate interpretation of all these findings for this endpoint and caution in interpretation is required. There was significant (*p* < 0.05) inter-study heterogeneity for certain endpoints for certain of these contrasts, a generally consistent feature of the analyses of leukaemia, brain/CNS tumours and thyroid cancer (including nodules) (Table 7). The *I*^2^ statistic was somewhat variable, generally near 0, but for brain/CNS tumours consistently above 50%, implying that a relatively large amount of the variance for this endpoint was accounted for by inter-study heterogeneity.

The analysis of Supplementary Tables S3 and S5, in which inverse-variance weighted linear models were employed to refit thyroid nodule data < 0.799 Gy of Hatch *et al* (Hatch et al. 2019), leukaemia data of Stevens *et al* (Stevens et al. 1990), and thyroid cancer data < 0.284 Gy of Kopecky *et al* (Kopecky et al. 2006) yielded generally similar findings, although the postnatal risk of leukaemia reduced but became (at least when using the lower dose set of risk estimates) highly significantly increasing (*p* < 0.001) (Supplementary Table S3). Additional analysis suggested that when using the lower dose study estimates there was highly significant heterogeneity in risk by endpoint (using the 6-endpoint split of Tables 6 and 7) overall (combining postnatal and *in utero* exposures) (*p* < 0.001), and also considering only studies of post-natal exposure (*p* < 0.001), but not for studies only of *in utero* exposure (*p* = 0.194) (results not shown). When using the higher dose study estimates there was highly significant heterogeneity in risk by endpoint overall (combining postnatal and *in utero* exposures) (*p* < 0.001), but not when considering only studies of postnatal exposure (*p* = 0.461), or for studies only of *in utero* exposure (*p* = 0.266) (results not shown). A complication with the analyses overall and for postnatal exposure only is that there were indications of non-convergence, whether using the lower or the higher risk estimates.

#### 3.3.2. Possible selection bias

Although the general symmetry of the funnel plots does not suggest any marked selection bias (Supplementary Figure S1), nevertheless the formal analysis of selection bias in Supplementary Table S8 implies that for many endpoints, in particular all postnatal endpoints, leukaemia, thyroid cancer (including nodules) and all cancer other than thyroid cancer (including nodules) there is significant selection bias (*p* < 0.05). However, the analysis of Supplementary Table S8 also demonstrates that adjusting for selection bias using the trim-and-fill method of Duval and Tweedie (Duval and Tweedie 2000) does not in general lead to marked changes in the central estimates, whether using the lower or higher set of risk estimates – only for lymphoma does the adjustment for selection bias lead to a marked reduction in ERR, although for the lower dose risk estimates the risk for all post-natal studies substantially increases after adjustment.

## 4. Discussion

### 4.1. General remarks

While understanding of the development of cancer at a cellular and sub-cellular level is very important and steadily increasing, and can inform risk extrapolation, epidemiology provides the human evidence most directly relevant to estimate radiation-related cancer risk. Because we are interested in the effects of low doses, large study populations are required to achieve sufficient statistical power. Studies of cancer after exposure in childhood have decisive advantages over studies of adult cancers: the frequency of cancer at young ages is relatively low and the ERR per unit dose is generally substantially higher than for exposure in adulthood (Grant et al. 2017; United Nations Scientific Committee on the Effects of Atomic Radiation (UNSCEAR) 2008; 2013). Most of the risk factors apart from radiation for the common childhood cancers are familial or genetic (Roman et al. 2018); there are few other risk factors that substantially modify risk at a population level in childhood (Linet et al. 2018). However, about 20% of childhood leukaemia could be attributed to natural background radiation on the basis of conventional risk estimates (Wakeford 2004; Wakeford et al. 2009).

The meta-analyses that we have undertaken combine studies that consider different ranges of attained ages. Many are of childhood cancers, but some extend into adulthood. We have attempted to allow for this in the analyses, in particular the analyses of exposures *in utero* (Supplementary Tables S4, S7), but this point should be borne in mind. In addition, as noted above, for many postnatal exposure endpoints (but not for studies of *in utero* exposure) a large proportion of the total variance is accounted for by inter-study heterogeneity, which complicates interpretation of the findings. Nevertheless, the meta-analysis of Table 6 using REML models suggests that there are significant risks for all cancers in relation to postnatal exposure (*p* < 0.01), with particularly large relative risks for brain/CNS tumours and leukaemia, whereas in relation to *in utero* exposure the strongest evidence of excess is for thyroid cancer (including nodules) (*p* = 0.033), although this is based on one study (Hatch et al. 2019). The meta-analysis using maximum-likelihood fitted models in Table 7 implies that for thyroid cancer (including nodules) there is significantly higher relative risk associated with *in utero* exposure compared with postnatal exposure. There are significant variations by dose and dose rate for certain endpoints, so that lung cancer relative risk appears to be highest for low dose-rate exposures, although the data are limited. There are suggestions of significant variation in relative risk between the cancer endpoints overall, but not when postnatal and *in utero* exposures are considered separately. The findings of selection bias in the data are somewhat troubling. Nevertheless, although statistically significant for a few endpoints (in particular leukaemia and thyroid), adjustment for such bias does not generally change the ERR estimates (Supplementary Table S8).

It is important to recognise that although relative risks for childhood exposure are relatively high, the absolute risks associated with exposure are quite low. For example, in the pooled analysis of leukaemia after childhood exposure the excess cases or deaths after 0.1 Gy were in the range 0.1–0.4 per 10,000 person-years of follow up (Little et al. 2018b). It is also important to recognize that there has been evidence for some time that relative risks associated with exposure *in utero* and in childhood are very likely not constant with attained age (Little et al. 1991). We have attempted to adjust for this at least in relation to the studies of *in utero* exposure (Supplementary Tables S5, S7), from which can be seen the substantial difference that is made. It is possible that such attained age effects could explain part of the heterogeneity observed in studies of postnatal exposure; unfortunately, it was less easy to get useful information on attained age in all of these studies.

### 4.2. Studies of atomic bomb survivors

The survivors of the Japanese atomic bombings offer a unique cohort which can throw light on low-dose effects as well as effects at higher doses – around two-thirds of the survivors received doses < 0.1 Gy. Cancer relative risks in the LSS (Table 4) are not very dissimilar in magnitude to those in diagnostically exposed groups (Table 2). There are suggestions that *in utero* relative risks in the atomic bomb survivors (Table 4) may be lower than those in some other groups, in particular, for endpoints such as lymphoma and brain/CNS tumours (Tables 2, 3, Supplementary Table S7), as is also indicated by the results of the meta-analysis (Table 7). It should be noted that there are limited numbers of cases and deaths among the *in utero* exposed occurring in childhood (age < 15 years), specifically one death from liver cancer (Delongchamp et al. 1997) and a non-fatal case of Wilms’ tumour (Yoshimoto et al. 1988). The first case of leukaemia, also the first death from this cause, occurred at age 18 years (Delongchamp et al. 1997; Yoshimoto et al. 1988).

For leukaemia there are indications of lower relative risk among those exposed *in utero* during the atomic bombings, with an average dose of ∼ 0.12 Gy (Sugiyama et al. 2021), than in the OSCC (Table 2) and in other case-control studies of intrauterine medical diagnostic exposure. This may be because of elevated sensitivity of the active bone marrow (ABM) to the competing effects of moderate doses of acutely delivered radiation *in utero*. It is notable that there are no leukaemia cases or deaths observed in childhood among the *in utero* exposed cohort, although the expected numbers are small (Delongchamp et al. 1997; Wakeford and Little 2003; Yoshimoto et al. 1988). The study of Ohtaki *et al* (Ohtaki et al. 2004) may have some bearing on this, as it suggested that stable chromosome translocations among the *in utero* exposed survivors exhibited a biphasic response, steeply increasing below about 0.1 Gy then involuting above that dose, indicating that haematopoietic cells may be damaged irreparably by moderate doses and replaced by viable cells.

Recent studies in the LSS have demonstrated that radiation incidence relative risk of female breast cancers is highest for exposure around menarche (Brenner et al. 2018) and that significantly increased radiation incidence risk of uterine corpus cancers is found only for exposure during the mid-pubertal period preceding menarche (Utada et al. 2019). During puberty, rapid stem cell proliferation of the terminal end buds mediates development of the mammary gland into a highly branched epithelial network (Scheele et al. 2017). Likewise, a dramatic increase in uterine volume and endometrial thickness occur during puberty prior to menarche (Hagen et al. 2015). A narrow age at exposure window for cancer radiosensitivity in the breast and uterine corpus may be related to radiation exposure during a period of such increased cell proliferation, and have significant implications for radiological protection.

### 4.3. Studies of in utero irradiation

The association between cancer in childhood and a prior radiographic examination of the abdomen of the pregnant mother identified by case-control studies such as those of Stewart *et al* (Bithell and Stewart 1975; Stewart et al. 1956) and many others (Wakeford 2008) (see Little *et al* (Little et al. 2021b, submitted)) provides epidemiological evidence that externally delivered doses of ionising radiation of the order of 0.005–0.030 Gy of X-rays increase the risk of cancer (United Nations Scientific Committee on the Effects of Atomic Radiation (UNSCEAR) 2008). This level of dose is somewhat lower than the lowest doses producing significantly increased risks of cancer in all other epidemiological studies, apart from the natural background radiation studies that we discuss below (United Nations Scientific Committee on the Effects of Atomic Radiation (UNSCEAR) 2008). Case-control studies have often been used in this setting, which can be subject to a number of biases, in particular selection, participation and recall biases, which can make them a poor choice for studies of medical exposure; however, with care, large case-control studies have been used in a medical setting without appreciable bias (MacMahon 1962). Nevertheless, the interpretation of the associations in this group of studies has been controversial. Doll and Wakeford (Doll and Wakeford 1997), after reviewing the available evidence, concluded that there are strong grounds for a causal interpretation of the association, and although this is still not universally accepted (International Commission on Radiological Protection (ICRP) 2003; National Council on Radiation Protection and Measurements (NCRP) 2013) there has been a degree of consensus in recent years (Armstrong et al. 2012; Wakeford and Little 2003) that this association may represent a cause-and-effect relationship.

As well as the medical diagnostic studies without assessed doses (which are reviewed in a separate paper (Little et al. 2021b, submitted)), there is information in a large number of studies of various exposed groups in which individual dose estimates are available (Tables 2, 3, Supplementary Table S7). One of the more intriguing findings of the meta-analysis are the indications of difference for certain endpoints in the magnitude of relative risk for exposures *in utero* and in the postnatal period (Tables 6, 7). In particular the analysis of Table 7 highlights the significant difference between these two types of exposure for thyroid cancer (including nodules), with relative risks tending to be higher for *in utero* than for postnatal exposure. Although not formally statistically significant there are also indications of much higher risks of lymphoma and brain/CNS associated with exposure *in utero* than for postnatal exposure (Table 7).

### 4.4. Studies of natural background radiation

As discussed by Kendall *et al* (Kendall et al. 2021), many of the early studies concentrated on effects of exposure from inhaled radon (an alpha-particle emitting noble gas) and its radioactive progeny, but as time went on, there was an increasing focus on studies of gamma radiation as well as, or instead of, radon, possibly driven by the realisation that doses from penetrating gamma radiation almost certainly accounted for most of the predicted radiation-related absolute risk of cancers in childhood (Kendall et al. 2021). A large number of studies of natural background radiation and childhood cancer have been conducted (Supplementary Table S6). The early studies tended to be ecological, but as time went on there was a preference for the more reliable case-control design. Insufficient statistical power is a particular problem for case-control studies, as realistically sized interview-based studies, with usually at most a few thousand cases, can never have high (>80%) statistical power to detect realistic excess risks (Little et al. 2010). As shown by Land (Land 1980) if a low power study produces a statistically significant positive trend it is almost always bound to be upwardly biased. Studies reporting positive associations are also more likely than negative ones to be written up and published, leading to a reporting bias. However, Hauptmann *et al* (Hauptmann et al. 2020) judged that most studies that are set up to study cancer and are of reasonable size would be published, whether null or not. Kendall *et al* (Kendall et al. 2021) discuss various other issues, in particular the problems of selection bias that may affect case-control studies. Largely in response to these two problems, of lack of statistical power and potential bias, a number of register-based national studies have been conducted over the last decade or so, and the advantages and disadvantages of these are discussed by Kendall *et al* (Kendall et al. 2021). Although free of selection, participation and recall bias, inevitably registry-based studies will lack individual dose estimates and many other individual covariates that can be collected by an interview-based case-control study. However, surrogates for some of these, for example indicators of socioeconomic status, are included in many datasets, and as noted above there are few large risks factors that operate at a population level for most childhood cancers. Therefore, this loss of information is perhaps not a large concern. There have been a number of European national register-based studies (Berlivet et al. 2021; Berlivet et al. 2020; Demoury et al. 2017; Kendall et al. 2013; Mazzei-Abba et al. 2021; Nikkilä et al. 2016; Spix et al. 2017; Spycher et al. 2015), recently reviewed by Mazzei-Abba *et al* (Mazzei-Abba et al. 2020) and by Kendall *et al* (Kendall et al. 2021). Most of these studies remain underpowered; only the British study (Kendall et al. 2013) and the French study (Berlivet et al. 2021; Demoury et al. 2017) have reasonable power, of 50% or more, to detect the predicted excess risk.

### 4.5. Studies of exposures due to man-made environmental contamination

In terms of population exposure, the Chernobyl reactor accident in northern Ukraine in 1986 was by far the largest nuclear accident, in particular, leading to significant intakes of internally deposited radio-active iodine among the populations of the former USSR (United Nations Scientific Committee on the Effects of Atomic Radiation (UNSCEAR) 2011). As noted in the Supplementary Methods, many thyroid doses and dose rates were very high, so that the screening studies of these populations, recently reviewed by Hatch and Cardis (Hatch and Cardis 2017) are outside the scope of the present review. The mean ABM dose from Chernobyl exposure, mainly external gamma radiation from deposited radiocaesium, was just 10 mGy and the study of childhood leukaemia in the Chernobyl-exposed populations has been problematical (Davis et al. 2006): there were concerns about the representativeness of the controls in the Ukrainian part of the study (Davis et al. 2006; Moysich et al. 2011), which led to a further case-control study being conducted in Ukraine that produced a substantially lower risk estimate (Noshchenko et al. 2010) (Table 5). The positive findings for thyroid cancer and benign neoplasms in the Nevada fallout study (Lyon et al. 2006) are also consistent with studies of exposures at slightly higher (but still low) levels of external dose (Lubin et al. 2017) (Tables 2, 5). They are also consistent with risks in the Chernobyl screening studies, although doses and dose rates here are high (Brenner et al. 2011; Little et al. 2014; Little et al. 2015). Both comparisons suggest that internal exposure or low dose rate exposure are unlikely to be the reason for the absence of thyroid cancer risks in the Hanford study (Davis et al. 2004). It is possible that the statistical power in the Hanford study is low.

The Fukushima Dai-ichi nuclear accident in Japan in 2011 also released radioactive iodine, but an order of magnitude less than the release during the Chernobyl accident, and thyroid doses were estimated to be much less than those received by children living around Chernobyl (United Nations Scientific Committee on the Effects of Atomic Radiation (UNSCEAR) 2014; 2021b). As yet, no studies of populations exposed from Fukushima have incorporated individual dosimetry; in particular this is the case for studies of thyroid cancer by Tsuda *et al* (Tsuda et al. 2016) and by Ohira *et al* (Ohira et al. 2020) (see Table 5); the study of Tsuda *et al* (Tsuda et al. 2016) has been much criticised on other grounds (Wakeford et al. 2016).

### 4.6. Studies of computed tomography (CT) in children

Large datasets of persons receiving substantial doses from CT examinations in childhood have been assembled (Bernier et al. 2019; Berrington de Gonzalez et al. 2016; Journy et al. 2015; Journy et al. 2016; Kojimahara et al. 2020; Krille et al. 2015; Mathews et al. 2013; Meulepas et al. 2019; Nikkilä et al. 2018; Pearce et al. 2012). In these CT studies there is some assessed potential for bias associated with reverse causation (Boice 2015; Walsh et al. 2014), that is that the CT scan might have been taken because of early symptoms from pre-existing (latent) disease and was therefore not a cause of the disease (Schubauer-Berigan et al. 2020; Walsh et al. 2014). *A priori* it is unlikely that reverse causation would have much role to play for leukaemia, as most cases are acute. Confounding by indication, in other words the possibility that high-risk conditions lead to increase in prevalence of CT imaging, is also a concern in these studies (Schubauer-Berigan et al. 2020; Walsh et al. 2014). Confounding by indication is quite distinct from reverse causation, although the two terms are often used interchangeably (Kummeling and Thijs 2008). Recent studies have demonstrated that although there is evidence of confounding by indication in the UK (Berrington de Gonzalez et al. 2016), French (Journy et al. 2015), Dutch (Meulepas et al. 2019) and Finnish (Nikkilä et al. 2018) studies, excluding patients with possibly predisposing syndromes (PS) did not much affect the trends with dose. A number of different methods were employed: in the UK study pathology reports from the cancer registries and radiologists’ notes were used to determine whether the cases and non-cases had any of a large number of PS (Berrington de Gonzalez et al. 2016); in the French study patient data from the hospital discharge data was used to diagnose patients with these PS, using a slightly smaller list (Journy et al. 2015); in the Dutch study only patients with tuberous sclerosis (one of the PS ascertained in the British but not in the French study) were ascertained via linkage with two hospitals treating most of the patients; and in the Finnish study (Nikkilä et al. 2018) only patients with Down syndrome were ascertained (one of the PS ascertained in the British and French studies). The theoretical study of Meulepas *et al.* examined plausible scenarios for confounding by indication, via inclusion of individuals with cancer susceptibility syndromes, and suggested that confounding by indication associated with inclusion of such persons was not expected to be substantial (Meulepas et al. 2016). Another theoretical study suggested that reverse causation is unlikely to result in bias away from the null for brain cancer (Little et al. 2021c in press). However, the study of Mathews *et al* (Mathews et al. 2013) suggests that care is required in the design and conduct of CT scan studies if confounding by indication is to be avoided.

### 4.7. Doses and dose rates in the studies considered and for in vivo radiobiological data

Doses from natural background radiation (Table 1, Supplementary Table S6), the medical *in utero* studies (Table 2) and the studies of fallout (Table 5) are exceptionally low (generally < 0.03 Gy), but in all studies considered here the dose rates are generally low or moderate – the highest dose rates were generally in the CT scan studies and in a study of persons receiving multiple fluoroscopic exposures as part of the monitoring of tuberculosis treatment (Little and Boice 1999), approach 0.1 Gy per hour; only in the study of Swedish children treated for skin haemangioma (Lundell et al. 1999) did dose rates greatly exceed 0.1 Gy per hour (Table 2). Natural background radiation exposures are ubiquitous and adequately sized cohorts can be assembled, though the studies need to be at a national or super-national scale in order to achieve reasonable statistical power (Little et al. 2010).

A particularly interesting finding is that relative risks associated with lung cancer were significantly lower in the moderate and high dose-rate studies than in the low dose-rate studies, also lower in the moderate and high dose studies than in the low dose studies (Table 7, Supplementary Table S5). This is not what would be expected conventionally, running somewhat counter to the general observation, based largely on radiobiological data, that cancer risks following low dose and low dose rate radiation would be below those at high dose rate (United Nations Scientific Committee on the Effects of Atomic Radiation (UNSCEAR) 1993; 2008). These results took no account of smoking data in the relevant datasets (Cahoon et al. 2017b; Ronckers et al. 2010), which could conceivably confound; nevertheless, analyses concentrating on lifelong smokers, or in which the baseline rates in the LSS were adjusted for smoking status and numbers of cigarettes per day smoked yielded ERR that were fairly close to the unadjusted ERR (Table 4). However, recent reanalysis of some large animal datasets did not yield very strong evidence for the ameliorating effects of low dose-rate or low dose exposure on cancer risk (Tran and Little 2017), although evidence of such dose rate effects is stronger when the less relevant endpoint of life shortening is used (Haley et al. 2015). This evidence relating to possible effects of dose rate is fairly weak, since we are comparing risks in moderate and high dose rate studies with those at low dose rate among very different study populations, with different periods of follow-up; nevertheless, what we have done is in the spirit of similar exercises that have been conducted in the epidemiological literature that attempt to assess dose rate effects (Hoel 2018; Jacob et al. 2009; Kocher et al. 2018; Little et al. 2021d; Shore et al. 2017; Walsh et al. 2021).

### 4.8. Other reviews of the literature

A group of NCI collaborators conducted a systematic review of 26 recently published epidemiological studies with mean doses<0.1 Gy (range 0.0001–0.082 Gy). These comprised eight environmental, four medical, and 14 occupational studies (Berrington de Gonzalez et al. 2020; Daniels et al. 2020; Gilbert et al. 2020; Hauptmann et al. 2020; Linet et al. 2020; Schubauer-Berigan et al. 2020). The review included six studies of cancer after childhood exposure, all included here. The review considered a critical appraisal of dosimetry methods, confounding and selection bias, outcome assessment problems and the effects of dose measurement errors. Meta-analysis was conducted of leukaemia risk after childhood exposure (Hauptmann et al. 2020). Both for solid cancers and for leukaemia, the majority of the studies reported positive ERRs per unit dose. Several limitations were identified, but only a few positive studies were potentially biased away from the null. These studies therefore directly supported excess cancer risks from low-dose ionising radiation (Berrington de Gonzalez et al. 2020; Daniels et al. 2020; Gilbert et al. 2020; Hauptmann et al. 2020; Linet et al. 2020; Schubauer-Berigan et al. 2020), and the magnitude of cancer risks was statistically compatible with those in the LSS (Hauptmann et al. 2020).

It is interesting to note how little overlap there is between the datasets considered in the NCI review (Hauptmann et al. 2020) and the present one, specifically six studies (Davis et al. 2006; Journy et al. 2015; Kendall et al. 2013; Lubin et al. 2017; Nikkilä et al. 2016; Spycher et al. 2015), although the version of the UK CT study used in the NCI review (Berrington de Gonzalez et al. 2016) is substantially the same as that employed here (Pearce et al. 2012). There is rather more overlap with the studies considered in a recent review by NCRP (National Council on Radiation Protection and Measurements (NCRP) 2018), specifically eleven studies (Akleyev et al. 2016; Demoury et al. 2017; Kendall et al. 2013; Krille et al. 2015; Little and Boice 1999; Lubin et al. 2017; Nikkilä et al. 2016; Pearce et al. 2012; Preston et al. 2008; Ronckers et al. 2008; Schüz et al. 2017). The fact that there is not greater overlap can be explained by a number of factors, specifically the fact that the NCI review omitted all studies published before 2006 and after 2017, and because of when it was done, the NCRP study (National Council on Radiation Protection and Measurements (NCRP) 2018) effectively did not include any studies published after 2017. The NCI study was limited to those datasets (excluding the Mayak workers and the Kerala natural background radiation studies) in which the mean dose was under 0.1 Gy (Hauptmann et al. 2020), unlike the present review, which limited coverage based on a combination of the maximum cumulative dose and maximum dose rate, so that studies which had dose rate > 0.1 Gy/hour and maximum dose > 1 Gy were excluded. The NCRP review was likewise limited to “a comprehensive review of recent (within ∼ 10 y) relevant epidemiologic studies with quantitative dose–response analyses” (National Council on Radiation Protection and Measurements (NCRP) 2018). Nevertheless, as inspection of Tables 1–5 demonstrates there are certain other studies that might have been included in previous reviews. For example, the lung cancer mortality part of the scoliosis study of Ronckers *et al* (Ronckers et al. 2010) and the German CT study of Krille *et al* (Krille et al. 2015) were both apparently eligible for inclusion in the NCI review, but were both excluded because of the abstract-based screening employed there. Likewise the mortality part of the scoliosis study of Ronckers *et al* (Ronckers et al. 2010) was apparently not considered for inclusion in the NCRP review. This highlights the difficulty of reviews based on automatic searches of databases such as PubMed, but may also reflect exclusions made in reviewing the searched articles using established criteria that were not made clear in the publications. Bearing on this it should be noted that a very large number of the papers we reviewed were not found in our PubMed search, but were found from assessments of other literature (see Supplementary Methods).

### 4.9. Biological data pertaining to the plausibility of low dose cancer risk

In this section we consider biological data that may support linearity of dose response for cancer, and why departures from linearity can be expected, as well as dose rate effects. The discussion here is mostly quite general, and is applicable to exposures at any age. Although curvature of dose response is not addressed in the low dose/low dose rate data considered in this paper, it is certainly observed at slightly higher levels of dose (United Nations Scientific Committee on the Effects of Atomic Radiation (UNSCEAR) 1993; 2008), and the presence of curvature is the reason why it is important that attention be paid to low dose or low dose rate effects – otherwise one would just consider the full dose range.

Cancer is thought to result from mutagenic damage to a single cell, specifically to its nuclear DNA, which in principle could be caused by a single radiation track, and this argues against the existence of a threshold of dose below which cancer risk is not elevated, as discussed elsewhere (Little et al. 2009). A more recent evaluation of the biological mechanisms relevant for low dose radiation cancer risk inference concluded that ‘*There remains good justification for the use of a nonthreshold model for risk inference for radiation protection purposes, given the present robust knowledge on the role of mutation and chromosomal aberrations in carcinogenesis’* and, in relation to the potential targets in addition to nuclear DNA, *‘The potential contributions of phenomena such as transmissible genomic instability, bystander phenomena, induction of abscopal effects and adaptive response remain unclear.’* (United Nations Scientific Committee on the Effects of Atomic Radiation (UNSCEAR) 2021a). A low LET radiation dose of 0.001 Gy corresponds to about one electron track hitting a cell nucleus (National Council on Radiation Protection and Measurements (NCRP) 2001; United Nations Scientific Committee on the Effects of Atomic Radiation (UNSCEAR) 1993). This suggests that at low doses (0.01 Gy or less spread over a year) it is unlikely that temporally and spatially separate electron tracks could cooperatively produce DNA damage (Brenner et al. 2003), so that in this very low dose region DNA damage at a cellular level would be proportional to dose. It is known that the efficiency of cellular repair processes varies with dose and dose rate (National Council on Radiation Protection and Measurements (NCRP) 2001; United Nations Scientific Committee on the Effects of Atomic Radiation (UNSCEAR) 1993), and this may be the reason for the curvature that is observed in the cancer dose response at higher levels of dose (e.g. for leukaemia (Hsu et al. 2013) and some solid cancers (Little et al. 2020)) and dose rate effects observed in epidemiological (United Nations Scientific Committee on the Effects of Atomic Radiation (UNSCEAR) 2008) and animal (Haley et al. 2015; Tran and Little 2017; United Nations Scientific Committee on the Effects of Atomic Radiation (UNSCEAR) 1993) data. DNA double strand breakage, and clustered damage (two or more lesions in close proximity) (United Nations Scientific Committee on the Effects of Atomic Radiation (UNSCEAR) 2012) are thought to be the most critical lesion induced by radiation (United Nations Scientific Committee on the Effects of Atomic Radiation (UNSCEAR) 1993). Repair of DNA double strand breaks (DSBs) relies on a number of pathways, even the most accurate of which, homologous recombination, is prone to errors (National Council on Radiation Protection and Measurements (NCRP) 2001); other repair pathways, e.g., non-homologous end joining, single-strand annealing, are intrinsically much more error prone (International Commission on Radiological Protection (ICRP) 2006; National Council on Radiation Protection and Measurements (NCRP) 2001). The variation in efficacy of repair that undoubtedly occurs will affect the magnitude of unrepaired and misrepaired damage and, whereas unrepaired damage is likely to result in cell death, non-lethal misrepaired damage by definition results in mutation. Some information is available on the age-dependence of induction of DNA and chromosomal damage, and while not many studies are available, any differences between the young and adults are not great (Gomolka et al. 2018; Oestreicher et al. 2018).

## 5. Conclusions

Here we have considered the overall question of the relationship between low-level exposure to low LET radiation in childhood and the consequent risk of cancer. Attention was mainly focused on leukaemia, the most common and best studied of the childhood cancers, but we have also presented evidence of excess risk of brain/CNS and thyroid cancer and thyroid nodule risk. The data presented here, particularly that in Tables 1 and 2, indicate that there is now little reasonable doubt that the childhood leukaemia risk extends into the low dose range conventionally considered to be doses<0.1 Gy of low LET radiation. We would suggest that the evidence for elevated leukaemia risk now extends down to 0.05 Gy, and indeed for acute lymphoblastic leukaemia, as shown in a recent pooling study of low dose studies, excess risk extends down to around 0.02 Gy (Little et al. 2018b). These studies (Little et al. 2018b) and studies of natural background radiation (Kendall et al. 2013; Mazzei-Abba et al. 2021; Spix et al. 2017; Spycher et al. 2015) and of medical diagnostic exposures (Berrington de Gonzalez et al. 2016; Journy et al. 2015; Journy et al. 2016; Krille et al. 2015; Mathews et al. 2013; Meulepas et al. 2019; Pearce et al. 2012) offer strong suggestions of excess risks for certain types of cancer at around the same level of dose, about 0.02 Gy. Studies should concentrate on all these endpoints (leukaemia, thyroid cancer and brain/CNS tumours), because in these the excess risk will be most clearly established, and at lower levels of dose, although it is likely that different patterns of low-dose response may emerge. Mechanistic understanding of the development of cancer at a cellular and molecular level makes such a possibility biologically plausible. Further integration of information, particularly quantitative information, on the biological mechanisms of radiation carcinogenesis and leukaemogenesis making use of the adverse outcome pathway framework (National Council on Radiation Protection and Measurements (NCRP) 2020) with the further refinement of epidemiological investigations such as those reviewed here will serve to characterise radiation-related cancer risks more precisely.

## Supplementary Material

1

## Figures and Tables

**Table 1:** Risks from childhood exposure in studies of natural background radiation.

Reference	Mean dose (Gy)	Max dose (Gy)^a^	Mean dose rate (Gy/h)	Max dose rate (Gy/h)^b^	Description of study data	Notes	Endpoint [incidence unless otherwise stated]	Number of cases / deaths	ERR / Gy (95% CI)

(Kendall et al. 2013)	0.0040	0.031 (L)	NA	1.597 × 10^−7^(L)	Great Britain NRCT study 1980–2006	Gamma only, mean equivalent ABM dose and range including dose from radon and gamma	Acute myeloid leukaemia Lymphoid leukaemia All leukaemia Brain/CNS All lymphoma NHL Hodgkin lymphoma	1316 7267 9058 6585 2319 983 939	40 (− 110,210) 100 (20,190) 90 (20,170) 20 (− 40,90) 10 (− 70,90) 40 (− 110,210) 40 (− 70,160)
(Spycher et al. 2015)	0.00906	0.0494 (L)	1.09 × 10^−7^	3.83 × 10^−7^ (L)	Swiss Cancer Registry study, 1990–2008, children < 16 y	Gamma only	Leukaemia Brain/CNS Lymphoma	530 423 328	36 (− 3,77) 42 (2,84) 7 (− 36,52)
(Nikkilô et al. 2016)	0.0019	>0.011 (L)	6.64 × 10^−8^	1.40 × 10^−7^ (L)	Finnish Cancer Registry study 1990–2011	median doses, dose rates in controls	Acute lymphoblastic leukaemia Acute myeloid leukaemia All leukaemia	786 101 937	− 10 (− 100,90) − 80 (− 250,150) − 30 (− 110,60)
(Demoury et al. 2017)	0.0158	0.0402 (L)	9.82 × 10^−8^	2.608 × 10^−7^ (L)	French Childhood Cancer Registry (RNCE) ecological study 1990–2009, and case-control study 2002–2007	mean, max dose and mean, max dose rate estimated from controls age 15 in Geocap case-control study	All acute leukaemia Acute lymphoblastic leukaemia Acute myeloid leukaemia	9056 7434 1465	0 (− 10,10) 10 (− 10,20) − 10 (− 30,20)
(Berlivet et al. 2020)	0.0063	0.0352 (L)	9.22 × 10^−8^	2.548 × 10^−7^ (L)	French Childhood Cancer Registry (RNCE) ecological study 2000–2012		Brain/CNS	5471	10 (− 6,26)
(Berlivet et al. 2021)	0.0047	0.0324 (L)	9.08 × 10^−8^	2.548 × 10^−7^ (L)	French Childhood Cancer Registry (RNCE) ecological study 1990–2009	Cumulative gamma dose	Acute leukaemia Acute lymphoblastic leukaemia BCP acute lymphoblastic leukaemia Acute myeloid leukaemia	6057 4982 4156 957	− 8 (− 26,14) − 4 (− 26,18) 2 (–22,32) − 12 (− 54,42)
						Cumulative ABM dose	Acute leukaemia Acute lymphoblastic leukaemia BCP acute lymphoblastic leukaemia Acute myeloid leukaemia	6057 4982 4156 957	− 10 (–22,4) − 6 (− 20,8) − 2 (− 18,14) − 16 (− 44,16)

CNS, central nervous system.

a (L) = maximum dose consistent with low dose, (M) = maximum dose consistent with moderate dose but not with low dose, (H) = maximum dose consistent with high dose but not with moderate or low dose.

b (L) = maximum dose rate consistent with low dose rate, (M) = maximum dose rate consistent with moderate dose rate but not with low dose rate, (H) = maximum dose rate consistent with high dose rate but not with moderate or low dose rate.

**Table 2:** Risks from childhood and *in utero* exposure in studies of medical diagnostic radiation exposure.

Reference	Mean dose (Gy)	Max dose (Gy)^a^	Mean dose rate (Gy/h)	Max dose rate (Gy/h)^b^	Description of study data	Notes	Endpoint [incidence unless otherwise stated]	Number of cases / deaths	ERR / Gy (95% CI)

**Postnatal exposure**									
(Pottern et al. 1990)	0.1275	0.55 (M)	NA	NA (M)	Lymphoid hyperplasia cohort treated 1938–1969 at Children’s Hospital Medical	Questionnaire data, mean dose weighted sum of exposed and unexposed	Thyroid nodule	54	64 (18,225)
	0.1376	0.53 (M)	NA	NA (M)	Center, Boston	Examination data, mean dose weighted sum of exposed and unexposed		81	7 (3,15)
(Little and Boice 1999)	1.2	5.4 (H)	∼0.01−0.1	0.1 (M)	Massachusetts TB fluoroscopy cohort, with exposure under age 20	Adjustment for attained age (centred at age 50)	Breast	78	0.062 (0.0005,0.93)
(Lundell et al. 1999)	0.09171	1 (M)	NA	<600 (H)	Swedish haemangioma cohort irradiated for skin haemangioma at Radiumhemmet Stockholm 1920–1959 and Sahlgrenska University Hospital Göteborg 1930–1965 and followed up 1958–1993	Refitted to data in paper < 1 Gy using a linear Poisson model, mean dose via breast year weighted mean, maximum dose rate from ( Lundell 1994)	Breast	215	−0.4 (−1.1,0.7)
(Ronckers et al. 2001)	0.004	0.03 (M)	NA	NA (M)	Dutch nasopharyngeal radium irradiated children treated for otitis serosa and frequency matched controls treated 1945–1981, followed 1982–1997		Lymphoproliferative and haematopoi eti c malignancy mortality	23	450 (50,1690)
(Ronckers et al. 2008)	0.121	1.11 (H)	0.0045	0.0156 (M)	US scoliosis cohort of women diagnosed with scoliosis 1912–1965 and followed (via questionnaire) up to 1992, given multiple diagnostic X-radiographs	Mean and maximum dose rate based on the mean and maximum breast dose per radiograph	Breast	78	2.9 (−0.1,8.6)
(Ronckers et al. 2010)	0.109	1.703 (H)	0.0045	0.0156 (M)	US scoliosis cohort of women diagnosed with scoliosis 1912–1965 and followed (via linkage with various registers) up to 2004, given multiple diagnostic X-radiographs	Mean and maximum dose rate based on the mean and maximum breast dose per radiograph, from (Ronckers et al. 2008)	Breast mortality	112	4.0 (1.0,9.4)
	0.047	0.447 (H)	0.001706	0.005913 (M)	US scoliosis cohort of women diagnosed with scoliosis 1912–1965 and followed (via linkage with various registers) from 1992 (date of questionnaire) to 2004, given multiple diagnostic X-radiographs	Mean and maximum dose rate based on the mean and maximum breast dose per radiograph, from (Ronckers et al. 2008), scaled by ratio between mean lung dose and mean breast dose	Lung mortality	17	−1.4 (−7.1,3.1)
(Pearce et al. 2012)	0.04463	>0.3302 (M)	NA	>0.044 (M)	UK-NCI paediatric CT cohort, age at first CT < 21 y, 1985–2008		Brain	135	23 (10, 49)
(Mathews et al. 2013)	0.0059	NA (M)	0.0046	NA (M)	Australian CT study of persons aged 0–19 years in 1/1985 or born after that point, 1985–2007	1 year lag, dose rates based on mean dose per scan	Leukaemia + MDS	246	39 (14,70)
	0.048	NA (M)	0.040	NA (M)		5 year lag, dose rates based on mean dose per scan	Brain/CNS	283	21 (14,29)
(Journy et al. 2015)	0.0069	>0.1 (M)	NA	NA (M)	French infant CT study, 2000–2011, age < 10 y at first CT, followed via RNCE	Median ABM dose - very similar to Journy *et al* (Journy et al. 2016) except this paper uses Poisson models with linear ERR model. 2 year exclusion	Leukaemia	17	47 (−65,159)
	0.0183	>0.1 (M)	NA	NA (M)		Median brain dose - very similar to Journy *et al* (Journy et al. 2016) except this paper uses Poisson models with linear ERR model. 4 year exclusion	Brain/CNS	13	−4 (−11,1)
	0.0069	>0.1 (M)	NA	NA (M)		Median ABM dose - very similar to Journy *et al* (Journy et al. 2016) except this paper uses Poisson models with linear ERR model. 2 year exclusion	Lymphoma	19	8 (−57,73)
(Krille et al. 2015)	0.0117	(M)	NA	NA (M)	German infant CT study, 1980–2010, age < 15 y at first CT, lag 2 y	Using ABM dose	Leukaemia	12	9 (−19,37)
	0.0344	(M)	NA	NA (M)	Using brain dose	Brain/CNS	7	8 (4,13)
(Berrington de Gonzalez et al. 2017)	0.012	0.689 (M)	NA	NA (M)	UK-NCI paediatric CT cohort, age at first CT < 22 y, 1980–2008	Using ABM dose, 2 y lag	Hodgkin lymphoma	65	−1 (−16,13)
(Lubin et al. 2017)	0.02991	0.2 (M)	NA	NA (M)	Pooled analysis of 9 datasets	Dose < 0.2 Gy	Thyroid cancer	252	11.1 (6.6,19.7)
	0.01730	0.1 (L)	NA	NA (M)		Dose < 0.1 Gy	Thyroid cancer	184	9.6 (3.7,17.0)
(Little et al. 2018b)	0.0196	0.1 (L)	NA	NA (M)	9 cohort pooled moderate dose medical + LSS analysis - dose < 0.1 Gy		Acute myeloid leukaemia + MDS	87	20.9(4.1,49.2)
							Acute lymphoblastic leukaemia	40	46.6 (3.5,187.1)
							Chronic myeloid leukaemia	36	−6.4 (<−10,13.6)
							Leukaemia excluding CLL	221	8.4 (−0.3,20.8)
(Little et al. 2018a)	0.0049	0.7949 (M)	NA	NA (M)	US Radiologic Technologist cohort, followed up via four questionnaires administered 1983–2014	Health endpoints and medical diagnostic exposure assessed via questionnaire. Risks for exposure aged 0–19 as part of model with separate windows for various age at exposure groups	Papillary thyroid cancer Thyroid cancer	275 414	6.75 (−3.36,23.24) 4.57 (−3.08,16.19)
(Nikkilä et al. 2018)	0.00629	0.0332 (L)	NA	0.0068 (M)	Finnish Cancer Registry based case–control study 1990–2011	median dose for controls, using NCICT software	Leukaemia	1093	130 (20,260)
(Meulepas et al. 2019)	0.0385	>0.22 (M)	NA	NA (M)	Dutch CT study of children (age < 18 y) at first CT, 1979–2012	Exclusion and lag 5 y, brain dose	Brain/CNS	84	8.6 (2.0,22.2)
	0.0095	>0.017 (L)	NA	NA (M)			Exclusion and lag 2 y, ABM dose	Leukaemia	44	2.1 (−1.2,24.0)
	0.0095	>0.017 (L)	NA	NA (M)	Exclusion and lag 2 y, ABM dose	Leukaemia + MDS	63	0.4 (−1.2,16.1)
(Kojimahara et al. 2020)	0.025	>0.032 (M)	NA	NA (M)	Case-control study of 120 cases and 360 appendicitis controls aged 10–24 y, 2011–2015	Mean dose to controls, lag 2 y, adjusted for parental education, history of neurological disease and ADD/ADHD	Brain/CNS Glioma	120 47	0 (−10,10) −10 (−30,10)
(Pasqual et al. 2020)	0.00002	0.217 (M)	NA	NA (M)	MOBI-Kids multinational case-control study of medical diagnostic radiation exposures among persons aged 10–24 y	Dose lag 2 y	Brain/CNS	844	0 (0,10)
(Stern et al. 2020)	0.029	0.75 (M)	NA	NA (M)	Cohort of children receiving catheterization at age < 1 y 1980–1998, linked to German Childhood Cancer Registry	Risk derived from given relative risk (and Cl) for > 0.094 Gy group by dividing (RR-1) by 0.094	All cancer	16	27.7 (−22.3,77.6)
(Zidane et al. 2021)	0.00198	0.1942 (M)	NA	NA (M)	French CATHY three-centre case-control study, with focus on childhood medical diagnostic procedures 2002–2006	Linear part of linear-quadratic-exponential dose response	Thyroid cancer	1071	17 (0.6,35)
***In utero* exposure**									
(Hagstrom et al. 1969)	∼0.1	∼0.15(M)	∼0.000065	∼0.000097 (L)	Women administered ^59^Fe in pregnancy at Vanderbilt University Hospital, 1945–1949 and followed up to 1967	Dose rate estimated via theoretical calculation based on physical half life of ^59^Fe, excess relative risk derived via dividing excess odds ratio by mean dose	Leukaemia mortality Lymphoma mortality Solid tumour mortality	1 1 2	+∞ (−9.74,+∞) +∞ (−9.74,+∞) +∞ (−8.08,+∞)
(Bithell and Stiller 1988)	NA	<0.03 (L)	NA	0.018 (M)	Oxford Survey of Childhood Cancers, case- control pairs born 1953–1972, estimated via jointly fitted log linear model of OR with log-linear model of dose based on UNSCEAR (United Nations Scientific Committee on the Effects of Atomic Radiation (UNSCEAR) 1972) estimates	Trimester 2, maximum dose rates from UNSCEAR (United Nations Scientific Committee on the Effects of Atomic Radiation (UNSCEAR) 1972) for 1943–1949 Trimester 3, maximum dose rates from UNSCEAR (United Nations Scientific Committee on the Effects of Atomic Radiation (UNSCEAR) 1972) for 1943–1949	All cancer mortality	8513	20.8 (0.27,61.8) 28.8 (17.1,43.6)
(Bithell 1993)	NA	<0.03 (L)	NA (L)	>0.0061 (M)	Oxford Survey of Childhood Cancers, case-control pairs born 1953–1979	Estimated via log linear-quadratic model of OR fitted to data of Gilman *et al* (Gilman et al. 1988) by year for 1959, using dose estimate of 6.1 mGy per obstetric radiograph of Mole ( Mole 1990) for that year	All cancer mortality	14,759	51 (28, 76)
(Pasqual et al. 2020)	0.00005	0.0127 (L)	NA	NA (M)	MOBI-Kids multinational case-control study of medical diagnostic radiation exposures among persons aged 10–24 y, OR of > 5 mGy vs < 5 mGy	Dose lag 0 y, ERR derived by dividing ERR for > 5 mGy vs 0–5 mGy by 0.005 (the mean dose difference between > 5 mGy and < 5 mGy groups must be between 0.00427 and 0.0127 Gy, and assumed to be ∼ 0.005 Gy given the skewed nature of the dose distribution)	Brain/CNS	844	70 (−96,502)

CNS, central nervous system.

a (L) = maximum dose consistent with low dose, (M) = maximum dose consistent with moderate dose but not with low dose, (H) = maximum dose consistent with high dose but not with moderate or low dose.

b (L) = maximum dose rate consistent with low dose rate, (M) = maximum dose rate consistent with moderate dose rate but not with low dose rate, (H) = maximum dose rate consistent with high dose rate but not with moderate or low dose rate.

**Table 3:** Risks resulting from *in utero* man-made environmental and maternal occupational exposure.

Reference	Mean dose (Gy)	Max dose (Gy)^a^	Mean dose rate (Gy/h)	Max dose rate (Gy/h)^b^	Description of study data	Notes	Endpoint (incidence unless otherwise stated)	Number of cases / deaths	ERR / Gy (95% CI)

(Akleyev et al. 2016)	0.0141	0.9449	NA	NA (L)	Techa River and Mayak Worker cohorts 1950–2009		Solid cancer (at an attained age up to 60 years)	369	− 2 (−4,1)
							Solid cancer mortality (at an attained age of up to 60 years)	196	− 2 (−6,1)
(Schüz et al 2017)	0.001	1.053	NA	NA (L)	Techa River and Mayak workers exposed/followed-up 1953–2009 (TR incidence), 1950–2009 (TR mortality), 1948–2009 (MW)	Median (among those without malignancy) rather than mean dose	Haematological malignancy (at an attained age of up to 61 years) Haematological malignancy mortality (at an attained age of up to 61 years)	58 36	7.7 (0.2,25.6) 1.6 (−0.9,11.9)
(Hatch et al. 2019)	0.0726 0.0726 0.05456	2.268 (H) 2.268 (H) 0.799(M)	0.0002602 0.0002602 0.0001955	0.008127 (M) 0.008127 (M) 0.002863 (L)	Ukraine *in utero* ^131^I exposed cohort	Risk as given Risk as given Refitted using binomial odds model using data from paper < 0.799 Gy Refitted using inverse-variance reweighted least squares model using data from paper < 0.799 Gy	Thyroid cancer (attained age 25–30 years) Thyroid nodule (benign and neoplastic or suspicious) (attained age 25–30 years)	8 241 237	3.91 (<−1.49, 65.66) 1.53 (0.22,3.59) 2.55 (0.66,5.36) 2.75 (1.92, 3.58)
(Johnson et al. 2008)	0.0002	0.0126 (L)	NA	NA (L)	US Radiologic Technologists offspring born 1921–1984	Refitted from published data via Poisson linear model	Leukaemia Lymphoma Solid cancer All cancer (childhood cancer < 20 years of age)	63 48 115 226	25.52 (−105.3,327.7) 229.1 (−36.17,823.9) 14.17 (90.45,213.2) 56.29 (−40.26,203.9)
(Bunch et al. 2009)	∼0.0007	>0.002 (L)	NA	NA (L)	Offspring of female members of UK National Registry for Radiation Workers	Refitted from published data via linear binomial odds model	Leukaemia and NHL Cancers other than leukaemia and NHL	2 5	0.00 (ࡊ1087, 41,320) 0.00 (ࡊ5.674 × 10^7^, 5.674 × 10^7^)

CNS, central nervous system.

a (L) = maximum dose consistent with low dose, (M) = maximum dose consistent with moderate dose but not with low dose, (H) = maximum dose consistent with high dose but not with moderate or low dose

b (L) = maximum dose rate consistent with low dose rate, (M) = maximum dose rate consistent with moderate dose rate but not with low dose rate, (H) = maximum dose rate consistent with high dose rate but not with moderate or low dose rate.

**Table 4:** Risks from postnatal and *in utero* exposure in the Japanese atomic bomb survivors.

Reference	Mean dose (Gy)	Max dose (Gy)^a^	Mean dose rate (Gy/h)	Max dose rate (Gy/h)^b^	Description of study data	Notes	Endpoint [incidence unless otherwise stated]	Number of cases / deaths	ERR / Gy (95% Cl)
**Postnatal exposure**									
(Preston et al. 2007)	0.05637	1 (M)	0.05637	1 (M)	LSS brain/CNS cancer incidence 1958–1998, DS02 brain dose	Refitted to downloadable data < 1 Gy brain dose, age < 20 y at exposure, via Poisson linear model, stratified by age at exposure, age, sex, city, distance category	Brain/CNS	103	0.85 (−0.54,3.69)
	0.01809	0.2 (M)	0.01809	0.2 (M)		Refitted to downloadable data < 0.2 Gy brain dose, age < 20 y at exposure, via Poisson linear model, stratified by age at exposure, age, sex, city, distance category		91	−0.76 (−4.21,7.80)
	0.01026	0.1 (L)	0.01026	0.1 (L)		Refitted to downloadable data < 0.1 Gy brain dose, age < 20 y at exposure, via Poisson linear model, stratified by age at exposure, age, sex, city, distance category		86	−3.56 (−9.21,12.33)
	0.05543	1 (M)	0.05543	1 (M)	LSS breast cancer incidence 1958–1998, DS02 breast dose	Refitted to downloadable data < 1 Gy breast dose, age < 20 y at exposure, via Poisson linear model, stratified by age at exposure, age, sex, city, distance category	Breast	425	−1.94 (0.87,3.42)
	0.01787	0.2 (M)	0.01787	0.2 (M)		Refitted to downloadable data < 0.2 Gy breast dose, age < 20 y at exposure, via Poisson linear model, stratified by age at exposure, age, sex, city, distance category		358	−1.81 (−1.27,6.38)
	0.009793	0.1 (L)	0.009793	0.1 (L)		Refitted to downloadable data < 0.1 Gy breast dose, age < 20 y at exposure, via Poisson linear model, stratified by age at exposure, age, sex, city, distance category		329	−2.39 (−6.91,4.82)
(Cahoon et al. 2017b)	0.05569	1 (M)	0.05569	1 (M)	LSS incidence 1958–2009, DS02R1 dose	Refitted to downloadable data < 1 Gy lung dose, age < 20 y at exposure, via Poisson linear model, stratified by age at exposure, age, sex, city, distance category	Lung	833	0.44 (−0.12,1.18)
	0.01782	0.2 (M)	0.01782	0.2 (M)		Refitted to downloadable data < 0.2 Gy lung dose, age < 20 y at exposure, via Poisson linear model, stratified by age at exposure, age, sex, city, distance category		759	3.74 (0.69,7.88)
	0.01028	0.1 (L)	0.01028	0.1 (L)		Refitted to downloadable data < 0.1 Gy lung dose, age < 20 y at exposure, via Poisson linear model, stratified by age at exposure, age, sex, city, distance category		711	6.57 (0.84,14.63)
	0.05569	1 (M)	0.05569	1 (M)		Refitted to downloadable data < 1 Gy lung dose, age < 20 y at exposure, via Poisson linear model, stratified by age at exposure, age, sex, city, distance category, adjusted for cigarette smoking status, cigarettes per day smoked		833	0.67 (0.30,1.15)
	0.01782	0.2 (M)	0.01782	0.2 (M)		Refitted to downloadable data < 0.2 Gy lung dose, age < 20 y at exposure, via Poisson linear model, stratified by age at exposure, age, sex, city, distance category, adjusted for cigarette smoking status, cigarettes per day smoked		759	0.41 (−0.15,1.13)
	0.01028	0.1 (L)	0.01028	0.1 (L)		Refitted to downloadable data < 0.1 Gy lung dose, age < 20 y at exposure, via Poisson linear model, stratified by age at exposure, age, sex, city, distance category, adjusted for cigarette smoking status, cigarettes per day smoked		711	1.64 (0.55,3.49)
	0.06851	1 (M)	0.05569	1 (M)		Refitted to downloadable data < 1 Gy lung dose, lifelong nonsmokers, age < 20 y at exposure, via Poisson linear model, stratified by age at exposure, age, sex, city, distance category		160	1.77 (0.29,4.36)
	0.01962	0.2 (M)	0.01782	0.2 (M)		Refitted to downloadable data < 0.2 Gy lung dose, lifelong nonsmokers, age < 20 y at exposure, via Poisson linear model, stratified by age at exposure, age, sex, city, distance category		140	5.94 (−1.06,20.73)
	0.01107	0.1 (L)	0.01028	0.1 (L)		Refitted to downloadable data < 0.1 Gy lung dose, lifelong nonsmokers, age < 20 y at exposure, via Poisson linear model, stratified by age at exposure, age, sex, city, distance category		131	
(Grant et al. 2017)	0.05415	1 (M)	0.05415	1 (M)	LSS incidence 1958’2009, DS02R1 dose	Refitted to downloadable data < 1 Gy colon dose, age < 20 y at exposure, via Poisson linear model, stratified by age at exposure, age, sex, city, distance category	Solid cancer	8308	0.90 (0.69,1.13)
	0.01767	0.2 (M)	0.01767	0.2 (M)		Refitted to downloadable data < 0.2 Gy colon dose, age < 20 y at exposure, via Poisson linear model, stratified by age at exposure, age, sex, city, distance category		7429	1.21 (0.40,2.09)
	0.01008	0.1 (L)	0.01008	0.1 (L)		Refitted to downloadable data < 0.1 Gy colon dose, age < 20 y at exposure, via Poisson linear model, stratified by age at exposure, age, sex, city, distance category		6994	2.08 (0.49,3.85)
***In utero* exposure**									
(Delongchamp et al. 1997)	0.01461	1 (M)	0.01461	1 (M)	LSS *in utero* mortality with DS86 doses, followed up to age 47 or 1997	Restricted to maternal uterine dose < 1 Gy, refitted via Poisson linear model stratified by sex, using published data. Upper profile bound uses Self and Liang (Self and Liang 1987) adjustment as MLE is on boundary	Leukaemia mortality	6	−1.33 (<−1.33,87.33)
						Restricted to maternal uterine dose < 1 Gy, refitted via Poisson linear model stratified by sex, using published data	Solid cancer mortality	60	0.25 (−1.24,5.35)
(Preston et al. 2008)	0.07329	1 (M)	0.07329	1 (M)	LSS *in utero* cohort, DS02 doses, followed to 1999 or age 55	Refitted to published data maternal uterine dose < 1 Gy via Poisson linear model	Solid cancer	91	1.45 (−0.04,3.88)
(Sugiyama et al. 2021)	0.07565	1 (M)	0.07565	1 (M)	LSS *in utero* mortality with DS02R1 doses, followed up 1950–2012, maternal uterine dose	Refitted to published data maternal uterine dose < 1 Gy via Poisson linear relative risk model, stratified by sex	Solid cancer mortality	132	− 0.41 (−1.34,0.93)
							Esophageal cancer mortality	8	1.21 (−2.23,16.09)
							Stomach cancer mortality	26	0.01 (−1.87,4.28)
							Colon cancer mortality	10	−0.13 (−2.40,7.95)
							Rectal cancer mortality	7	1.40 (−2.20,17.12)
							Liver cancer mortality	18	− 2.10 (−2.76,0.65)
							Pancreas cancer mortality	7	1.40 (−2.20,16.78)
							Lung cancer mortality	21	−1.40 (−2.58,2.26)
							Lymphohaematopoietic mortality	8	4.45 (−1.90,60.14)
							Leukaemia mortality	5	− 2.80 (−2.84,14.22)

CNS, central nervous system.

a (L) = maximum dose consistent with low dose, (M) = maximum dose consistent with moderate dose but not with low dose, (H) = maximum dose consistent with high dose but not with moderate or low dose.

b (L) = maximum dose rate consistent with low dose rate, (M) = maximum dose rate consistent with moderate dose rate but not with low dose rate, (H) = maximum dose rate consistent with high dose rate but not with moderate or low dose rate.

**Table 5:** Risks resulting from postnatal man-made environmental exposure.

Reference	Mean dose (Gy)	Max dose (Gy)^a^	Mean dose rate (Gy/h)	Max dose rate (Gy/h)^b^	Description of study data	Notes	Endpoint [incidence unless otherwise stated]	Number of cases / deaths	ERR / Gy (95% Cl)

(Stevens et al. 1990)	0.0032	0.026 (L)	NA	NA (L)	Utah fallout and leukaemia case-control study 1952–1981	Linear binomial odds model fitted to data from paper. Median dose for all cases and controls used as mean	All leukaemia Chronic lymphocytic leukaemia (CLL) Leukaemia excluding CLL	1177 238 939	29.47 (−1.33,72.21) 48.55 (−16.47,187.70) 24.54 (−7.88,71.26)
						Inverse-variance weighted linear model fitted to data from paper. Median dose for all cases and controls used as mean	All leukaemia Chronic lymphocytic leukaemia Leukaemia excluding CLL	1177 238 939	184.13 (−718.10,1086.35) 187.52 (−662.10,1037.14) 187.80 (−693.92,1069.51)
(Parkin et al. 1996)	~7.8 × 10^−5^	>0.0003 (L)	NA	NA (L)	Ecologic analysis of childhood leukaemia in 23 European countries after Chernobyl nuclear accident 1980–1991	ERR / Gy derived via ERR/Sv ± 1.96 SE. Mean	Leukaemia	25,820	−22.20 (−84.92,40.52)
(Davis et al. 2004)	0.174	2.823 (H)	NA	NA (L)	Hanford cohort study of sampled births 1940–1946 in eastern Washington State, followed to 1997		Thyroid cancer Benign thyroid nodules	19 249	0.00 (<−0.001,0.02) − 0.01 (<−0.022,0.04)
(Cardis et al. 2005)	0.2720 0.3337 0.3598	1 (M) 1.5 (H) 2 (H)	0.000975 0.001196 0.001289	0.00358 (L) 0.005375 (M) 0.007167 (M)	Belarus & Russia thyroid cancer case-control study, 1992–1998 after ^131^I exposure from Chernobyl, persons aged <15 y at time of accident	Linear model <1 Gy, OR at 1 Gy Log-linear model <1 Gy, OR at 1 Gy Linear model <1.5 Gy, OR at 1 Gy Log-linear model <1.5 Gy, OR at 1 Gy Linear model <2 Gy, OR at 1 Gy Log-linear model <2 Gy, OR at 1 Gy	Thyroid cancer	218 218 242 242 252 252	6.6 (2.0,11.1) 8.4 (4.1,17.3) 5.8 (2.1,9.4) 5.9 (3.3,10.5) 5.5 (2.2,8.8) 5.5 (3.1,9.5)
(Davis et al. 2006)	0.01174	0.18619 (M)	NA	NA (L)	Belarus part of three country (Belarus, Russia and Ukraine) case-control study 1986–2000, children aged <6 y or *in utero* at time of Chernobyl accident	Mean dose among controls	Leukaemia	114	4.09 (NA, 37.7)
	0.01049	0.20238 (M)	NA	NA (L)	Russian part of three country (Belarus, Russia and Ukraine) case-control study 1986–2000, children aged <6 y or *in utero* at time of Chernobyl accident	Mean dose among controls, using log-linear model		39	− 2.7 (−31.5, 26.1)
(Kopecky et al. 2006)	0.016 0.0089	2.73 (H) 0.284 (M)	0.000057 0.000032	0.009782 (M) 0.001018 (L)	Bryansk case-control study 1986–1998, persons aged <20 y at time of Chernobyl accident	Median dose for controls used as mean Median dose in middle dose group used for mean, analysis restricted to <0.284 Gy, refitted by linear binomial odds model to data from paper Median dose in middle dose group used for mean, analysis restricted to <0.284 Gy, refitted by inverse-variance weighted linear model to data from paper	Thyroid cancer	66 45	138.00 (−0.36,>10^6^) 4.57 (−5.95,29.34) 31.81 (−194.77, 258.40)
(Lyon et al. 2006)	0.12	>0.41 (M)	NA	NA (L)	Utah, Nevada, Arizona fallout cohort study 1965–1998 among persons aged 12–18 y in 1965–1966, Phase IIR analysis		Thyroid cancer Thyroid neoplasms Thyroid nodule	8 20 49 246	0.8 (0.0,14.9) 13.02 (2.70,68.70) 4.65 (1.10,12.30)
(Noshchenko et al. 2010)	0.003635	0.3136 (M)	NA	NA (L)	Study cases among children (age 0-5 years at the time of accident) of four most highly Chernobyl-contaminated regions (oblasts) of Ukraine diagnosed 1987–1997	Mean dose in controls, linear part of dose response used for regression coefficient	Leukaemia	246	1.3 (1.0,1.7)
(Akleyev et al. 2016)	0.0112 0.057	0.552 (M) >0.220 (M)	NA NA	NA (L) NA (L)	Techa River and Mayak Worker *in utero* + postnatal cohorts 1950–2009 [postnatal exposure]	Stomach dose used Person year weighted mean dose	Solid cancer Solid cancer mortality	369 196 58	2 (0, 4) 2 (−1, 5) 2.1 (−0.5,11.0)
(Schüz et al 2017)		>0.221 (M)			Techa River & Mayak Worker *in utero* + postnatal cohort study, 1953–2009 (TR)+ 1948–2009 (MW) [postnatal exposure]		Haematologi cal malignancy Haematologi cal malignancy mortality	36	0.8 (−0.5,7.2)
(Ohira et al. 2020)	0.02549	0.058 (L)	NA	NA (L)	Fukushima cohort study 2011–2017, ages 6–14, aged < 18 y at time of Fukushima accident	Inverse-variance weighted linear model refitted to RR data, adjusted for age, sex, examination year, by dose group from paper. Mean dose estimated via midpoint of each dose group weighted by persons	Thyroid cancer	45	9.94 (−2.73,22.60)

CNS, central nervous system.

a (L) = maximum dose consistent with low dose, (M) = maximum dose consistent with moderate dose but not with low dose, (H) = maximum dose consistent with high dose but not with moderate or low dose.

b (L) = maximum dose rate consistent with low dose rate, (M) = maximum dose rate consistent with moderate dose rate but not with low dose rate, (H) = maximum dose rate consistent with high dose rate but not with moderate or low dose rate.

**Table 6:** Restricted maximum likelihood (REML) and DerSimonian and Laird 1-step random effects model fits to various subsets (as given by Supplementary Table S2).^a^

Endpoint	Excess relative risk (ERR) / Gy (95% CI)	*p*-value	Residual heterogeneity *p*-value	*I*^2^ (%)

**Analysis using lower dose risk estimates^b^** Postnatal exposure				
Leukaemia	5.20 (− 1.13,11.53)^c^	0.108^c^	0.027^c^	44.13^c^
Lymphoma (including CLL)	1.21 (−11.96,14.38)	0.857	0.899	0.00
Brain/CNS	6.81 (0.58,13.04)	0.032	<0.001	78.57
Lung	2.23 (−5.55,10.02)	0.574	0.069	69.87
Thyroid (including nodules)	0.00 (−0.07,0.08)^c^	0.928^c^	<0.001^c^	65.64^c^
All solid except brain/CNS, lung, thyroid	0.00 (−0.41,0.42)	0.983	0.189	0.00
All six endpoints only^d^	0.23 (0.07,0.39)^c^	0.006^c^	<0.001^c^	72.61^c^
All endpoints^d^	0.26 (0.10,0.43)^c^	0.001^c^	<0.001^c^	73.90^c^
*In utero* exposure				
Leukaemia	− 2.70 (−11.07,5.67)	0.527	0.966	0.00
Lymphoma (including CLL)	229.10 (−200.94,659.14)	0.296	1.000	0.00
Brain/CNS	70.00 (−229.00,369.00)	0.646	1.000	0.00
Lung	−1.40 (−3.82,1.03)	0.258	1.000	0.00
Thyroid (including nodules)	2.55 (0.20,4.90)	0.033	0.937	0.00
All solid except brain/CNS, lung, thyroid	−1.09 (−2.75,0.56)	0.196	0.754	9.23
All six endpoints only^d^	−0.26 (−1.92,1.39)	0.757	0.478	29.53
All endpoints^d^	0.96 (− 1.12,3.04)^c^	0.365^c^	0.004^c^	58.84^c^
**Analysis using higher dose risk estimates** ^e^				
Postnatal exposure				
Leukaemia	5.20 (− 1.13,11.53)^c^	0.108^c^	0.027^c^	44.13^c^
Lymphoma (including CLL)	1.21 (−11.96,14.38)	0.857	0.899	0.00
Brain/CNS	6.87 (1.02,12.72)	0.021	<0.001	85.15
Lung	0.41 (−0.23,1.06)	0.209	0.483	0.00
Thyroid (including nodules)	0.01 (−0.08,0.09)^c^	0.874^c^	<0.001^c^	73.37^c^
All solid except brain/CNS, lung, thyroid	0.77 (−0.61,2.14)	0.274	0.007	83.57
All six endpoints only^d^	0.28 (0.12,0.44)^c^	<0.001^c^	<0.001^c^	74.85^c^
All endpoints^d^	0.41 (0.24,0.58)^c^	<0.001^c^	<0.001^c^	80.79^c^
*In utero* exposure				
Leukaemia	− 2.70 (−11.07,5.67)	0.527	0.966	0.00
Lymphoma (including CLL)	229.10 (−200.94,659.14)	0.296	1.000	0.00
Brain/CNS	70.00 (−229.00,369.00)	0.646	1.000	0.00
Lung	−1.40 (−3.82,1.03)	0.258	1.000	0.00
Thyroid (including nodules)	1.54 (−0.15,3.22)	0.074	0.890	0.00
All solid except brain/CNS, lung, thyroid	−1.09 (−2.75,0.56)	0.196	0.754	9.23
All six endpoints only^d^	−0.38 (−1.87,1.10)	0.613	0.550	26.44
All endpoints^d^	0.70 (− 1.17,2.57)^c^	0.463^c^	0.006^c^	56.70^c^

a using binomial odds model to refit thyroid nodule data < 0.799 Gy of Hatch *et al* (Hatch et al. 2019) and leukaemia data of Stevens *et al* (Stevens et al. 1990), and inverse-variance weighted linear model to refit thyroid cancer data < 0.284 Gy of Kopecky *et al* (Kopecky et al. 2006)

b using refitted thyroid nodule data < 0.799 Gy of Hatch *et al* (Hatch et al. 2019), thyroid cancer data < 0.284 Gy of Kopecky *et al* (Kopecky et al. 2006), Cardis *et al* (Cardis et al. 2005) thyroid cancer data using a linear model restricted to < 1 Gy, Lubin *et al* (Lubin et al. 2017) data restricted to < 0.1 Gy, Preston *et al* (Preston et al. 2007) brain/CNS and breast cancer data restricted to < 0.1 Gy, Cahoon *et al* (Cahoon et al. 2017b) lung cancer data restricted to < 0.1 Gy.

c indications of non-convergence for REML model so that 1-step random effects model of DerSimonian and Laird (DerSimonian and Laird 1986) was employed instead.

d for “all endpoints” all endpoints were considered, whereas for “all six endpoints” the endpoint considered within a particular study had to lie within one of the six specified endpoints shown.

e using full range thyroid nodule data of Hatch *et al* (Hatch et al. 2019), thyroid cancer data of Kopecky *et al* (Kopecky et al. 2006), Cardis *et al* (Cardis et al. 2005) thyroid cancer data using a linear model restricted to < 2 Gy, Lubin *et al* (Lubin et al. 2017) data restricted to < 0.2 Gy, Preston *et al* (Preston et al. 2007) brain/CNS and breast cancer data restricted to < 1 Gy, Cahoon *et al* (Cahoon et al. 2017b) lung cancer data restricted to < 1 Gy.

**Table 7:** Maximum likelihood fits to assess significance of improvement in fit of various explanatory variables (to data as specified by Supplementary Table S2).^a^

**Analysis of *in utero* vs postnatal exposure**					
Lower dose risk estimates^b^					
Endpoint	ERR / Gy (95% CI) *(in utero* dose)	ERR / Gy (95% CI) (postnatal dose)	*p*-value (improvement in fit over null = no difference)	Residual heterogeneity *p*-value	*I* ^2^
Leukaemia	− 2.70 (−11.07,5.67)	1.31 (0.96,1.66)	0.348	0.052	0.00
Lymphoma (including CLL)	229.10 (−200.94,659.14)	1.21 (−11.96,14.38)	0.299	0.899	0.00
Brain/CNS	70.00 (−229.37,369.37)	6.65 (0.80,12.49)	0.678	<0.001	75.53
Lung	−1.40 (−3.82,1.03)	1.42 (−2.68,5.52)	0.247	0.069	0.00
Thyroid (including nodules)	2.55 (0.20,4.90)^c^	0.00 (−0.01,0.01)^c^	0.033^c^	0.001^c^	0.00^c^
All solid except brain/CNS, lung, thyroid	−1.31 (−2.70,0.07)	0.00 (−0.41,0.42)	0.074	0.492	0.00
Higher dose risk estimates^d^					
Endpoint	ERR / Gy (95% CI) *(in utero* dose)	ERR / Gy (95% CI) (postnatal dose)	*p*-value (improvement in fit over null = no difference)	Residual heterogeneity *p*-value	*I* ^2^
Leukaemia	− 2.70 (−11.07,5.67)	1.31 (0.96,1.66)	0.348	0.052	0.00
Lymphoma (including CLL)	229.10 (−200.94,659.14)	1.21 (−11.96,14.38)	0.299	0.899	0.00
Brain/CNS	70.00 (−229.33,369.33)	6.66 (1.20,12.13)	0.678	<0.001	82.79
Lung	−1.40 (−3.82,1.03)	0.41 (−0.23,1.06)	0.157	0.483	0.00
Thyroid (including nodules)	1.54 (−0.15,3.22)^c^	0.00 (−0.01,0.01)^c^	0.074^c^	<0.001^c^	0.00^c^
All solid except brain/CNS, lung, thyroid	−1.08 (−2.75,0.59)	0.57 (−0.37,1.51)	0.122	0.065	44.03
**Analysis of low dose studies vs moderate + high dose studies (using L/M/H coding of maximum dose in column 3 of** Tables 1–5)					
Lower dose risk estimates^b^					
Endpoint	ERR / Gy (95% CI) (low dose)	ERR / Gy (95% CI) (moderate + high dose)	*p*-value (improvement in fit over null = no difference)	Residual heterogeneity *p*-value	*I* ^2^
Leukaemia	1.74 (−3.64,7.11)	1.30 (0.95,1.65)	0.874	0.042	0.00
Lymphoma (including CLL)	14.31 (−21.63,50.25)	−0.57 (−14.73,13.58)	0.450	0.813	0.00
Brain/CNS	6.49 (−6.62,19.60)	6.71 (0.19,13.23)	0.977	<0.001	74.26
Lung	6.57 (−0.32,13.46)	−1.40 (−3.58,0.79)	0.031	0.999	0.00
Thyroid (including nodules)	9.67 (3.79,15.56)^c^	0.00 (−0.01,0.01)^c^	0.001^c^	0.010^c^	0.00^c^
All solid except brain/CNS, lung, thyroid	− 2.39 (−8.25,3.48)	−0.09 (−0.49,0.30)	0.444	0.264	0.00
Higher dose risk estimates^d^					
Endpoint	ERR / Gy (95% CI) (low dose)	ERR / Gy (95% CI) (moderate + high dose)	*p*-value (improvement in fit over null = no difference)	Residual heterogeneity *p*-value	*I* ^2^
Leukaemia	1.74 (−3.64,7.11)	1.30 (0.95,1.65)	0.874	0.042	0.00
Lymphoma (including CLL)	14.31 (−21.63,50.25)	−0.57 (−14.73,13.58)	0.450	0.813	0.00
Brain/CNS	16.63 (−1.55,34.81)	5.67 (0.08,11.26)	0.260	<0.001	81.66
Lung	NA	NA	NA	NA	NA
Thyroid (including nodules)	9.94 (−2.73,22.60)^c^	0.00 (−0.01,0.01)^c^	0.124^c^	<0.001^c^	0.00^c^
All solid except brain/CNS, lung, thyroid	NA	NA	NA	NA	NA
**Analysis of low dose rate studies vs moderate + high dose rate studies (using L/M/H coding of maximum dose rate in column 5 of** Tables 1–5)					
Lower dose risk estimates^b^					
Endpoint	ERR / Gy (95% CI) (low dose rate)	ERR / Gy (95% CI) (moderate + high dose rate)	*p*-value (improvement in fit over null = no difference)	Residual heterogeneity *p*-value	*I* ^2^
Leukaemia	1.30 (0.95,1.65)	1.03 (−3.99,6.06)	0.917	0.042	0.00
Lymphoma (including CLL)	14.31 (−21.63,50.25)	−0.57 (−14.73,13.58)	0.450	0.813	0.00
Brain/CNS	6.36 (−6.75,19.48)	6.74 (0.23,13.25)	0.960	<0.001	74.22
Lung	6.57 (−0.32,13.46)	−1.40 (−3.58,0.79)	0.031	0.999	0.00
Thyroid (including nodules)	0.00 (−0.01,0.01)^c^	7.97 (4.06,11.88)^c^	<0.001^c^	0.057^c^	0.00^c^
All solid except brain/CNS, lung, thyroid	− 2.39 (−8.25,3.48)	−0.09 (−0.49,0.30)	0.444	0.264	0.00
Higher dose risk estimates^d^					
Endpoint	ERR / Gy (95% Cl) (low dose rate)	ERR / Gy (95% Cl) (moderate + high dose rate)	*p*-value (improvement in fit over null = no difference)	Residual heterogeneity *p*-value	*I^2^*
Leukaemia	1.30 (0.95,1.65)	1.03 (−3.99,6.06)	0.917	0.042	0.00
Lymphoma (including CLL)	14.31 (−21.63,50.25)	−0.57 (−14.73,13.58)	0.450	0.813	0.00
Brain/CNS	16.43 (−1.78,34.64)	5.69 (0.10,11.28)	0.271	<0.001	81.65
Lung	NA	NA	NA	NA	NA
Thyroid (including nodules)	0.00 (−0.01,0.01)^c^	3.15 (1.75,4.55)^c^	<0.001^c^	0.030^c^	0.00^c^
All solid except brain/CNS, lung, thyroid	NA	NA	NA	NA	NA

a using binomial odds model to refit thyroid nodule data < 0.799 Gy of Hatch *et al* (Hatch et al. 2019) and leukaemia data of Stevens *et al* (Stevens et al. 1990), and inverse-variance weighted linear model to refit thyroid cancer data < 0.284 Gy of Kopecky *et al* (Kopecky et al. 2006)

b using refitted thyroid nodule data < 0.799 Gy of Hatch *et al* (Hatch et al. 2019), thyroid cancer data < 0.284 Gy of Kopecky *et al* (Kopecky et al. 2006), Cardis *et al* (Cardis et al. 2005) thyroid cancer data using a linear model restricted to < 1 Gy, Lubin *et al* (Lubin et al. 2017) data restricted to < 0.1 Gy, Preston *et al* (Preston et al. 2007) brain/CNS and breast cancer data restricted to < 0.1 Gy, Cahoon *et al* (Cahoon et al. 2017b) lung cancer data restricted to < 0.1 Gy.

c indications of non-convergence for maximum likelihood fitted model.

d using full range thyroid nodule data of Hatch *et al* (Hatch et al. 2019), thyroid cancer data of Kopecky *et al* (Kopecky et al. 2006), Cardis *et al* (Cardis et al. 2005) thyroid cancer data using a linear model restricted to < 2 Gy, Lubin *et al* (Lubin et al. 2017) data restricted to < 0.2 Gy, Preston *et al* (Preston et al. 2007) brain/CNS and breast cancer data restricted to < 1 Gy, Cahoon *et al* (Cahoon et al. 2017b) lung cancer data restricted to < 1 Gy.
