# Supplementary material for: Review of the risk of cancer following low and moderate doses of sparsely ionising radiation received in early life in groups with individually estimated doses
Source: Environ Int. Author manuscript; Available in PMC 2022 May 19. (PMC9118883; doi:10.1016/j.envint.2021.106983)
Supplement: 1 [file NIHMS1763948-supplement-1.docx]

**Supplement**

**Supplementary Methods**

*S.1 Literature review*

A literature search of PubMed was performed on 16^th^ May 2021 using the search terms:

(cancer*[tiab] OR malignan*[tiab] OR tumour*[tiab] OR tumor*[tiab])

AND ("X ray"[tiab] OR "X-ray"[tiab] OR "x-ray"[tiab] OR radiation*[tiab] OR radionuclide*[tiab] OR radioisotope*[tiab] OR "radioactive nuclide*"[tiab] OR "radioactive isotope*"[tiab] OR "CT scan*"[tiab] OR “CT-scan*”[tiab] OR "computed tomograph*"[tiab] OR "computerized tomograph*"[tiab] OR "computerised tomograph*"[tiab] OR fluoroscop*[tiab] OR radiograph*[tiab] OR angiograph*[tiab] OR cardiograph*[tiab] OR “cardiac catheter*”[tiab] OR “interventional cardiol*”[tiab] OR “interventional radiograph*”[tiab] OR radiol*[tiab] OR "atomic bomb"[tiab] OR Hiroshima[tiab] OR Nagasaki[tiab] OR Chernobyl[tiab] OR Chornobyl[tiab] OR Fukushima[tiab] OR "nuclear accident*"[tiab] OR "nuclear reactor*"[tiab])

AND (childhood*[tiab] OR "in utero"[tiab] OR obstetric[tiab] OR pregnancy[tiab])

NOT radiotherapy[tiab] NOT "radiation therapy"[tiab] NOT "chemotherapy"[tiab] NOT "roentgen therapy"[tiab] NOT "roentgen treatment"[tiab] NOT letter[ptyp] NOT editorial[ptyp] NOT comment[ptyp] NOT news[ptyp] NOT "Congress"[Publication Type] NOT "Consensus Development Conference"[Publication Type] NOT editorial[tiab] NOT commentary[tiab] NOT “conference abstract*”[tiab] NOT “conference proceeding*”[tiab] NOT “systematic review*”[ti] NOT “meta-analysis”[ptyp] NOT “meta-analysis”[ti] NOT “meta-analyses”[ti] NOT "Review"[Publication Type] NOT "Systematic Review"[Publication Type] NOT “retracted publication”[ptyp] NOT “retraction of publication”[ptyp] NOT

“retraction of publication”[tiab] NOT “retraction notice”[ti] NOT “retracted publication”[tiab] NOT "Published Erratum"[Publication Type] NOT Corrigenda[tiab] NOT corrigendum[tiab] NOT errata[tiab] NOT erratum[tiab] NOT protocol[ti] NOT protocols[ti]

NOT animals[tiab] NOT animal*[tiab] NOT mice[tiab] NOT mouse[tiab] NOT rat[tiab] NOT rats[tiab] NOT dog[tiab] NOT dogs[tiab] NOT pig[tiab] NOT pigs[tiab] NOT swine[tiab] NOT porcine*[tiab] NOT rodent*[tiab]

A total of 3117 papers were returned. A PECO statement is given in Supplementary Table S9. The titles and abstracts of these were independently double scanned by MPL and GMK, and case reports, review papers and other clearly inapplicable results (e.g. relating to populations not exposed in childhood) were eliminated. Consistency was established via consensus. Additionally, recent UNSCEAR reports (United Nations Scientific Committee on the Effects of Atomic Radiation (UNSCEAR) 2008; 2013; 2018) were scanned to assess additional literature, as well as recent review articles (Kendall et al. 2021; Wakeford and Bithell 2021). A total of 299 papers that were deemed applicable based on the title/abstract search, and the associated full publications were then obtained for more detailed review of these by MPL and GMK.

Of the 299 consensus sample we restricted attention to those studies of persons exposed *in utero* or in childhood (age 20y or less) either with individually estimated organ/tissue doses and either with maximum cumulative doses (or if this could not be determined, mean cumulative doses) not exceeding the conventional definitions of low doses, <0.1 Gy, or moderate doses, 0.1-1 Gy (Harrison et al. 2021; Little et al. 2021a), or in which the maximum dose rate did not exceed 0.005 Gy per hour (the conventional upper limit for low dose rate (United Nations Scientific Committee on the Effects of Atomic Radiation (UNSCEAR) 2020)) or 0.1 Gy per hour (which we take as the upper limit for moderate dose rate), or in which the maximum cumulative dose (or if this could not be determined the mean cumulative dose) did not exceed 0.1 Gy (the low dose range) or 1 Gy (the moderate dose range). The levels of dose used have been previously employed (Little et al. 2021a; United Nations Scientific Committee on the Effects of Atomic Radiation (UNSCEAR) 2015). ICRP have determined that “when dose rates are lower than around 0.1 Gy/hour there is repair of cellular radiation injury during the irradiation” (International Commission on Radiological Protection (ICRP) 2007), suggesting that 0.1 Gy per hour is a reasonable limit for moderate dose-rate irradiation. Again, consistency between the reviewers was established via consensus; all studies that had been superseded by others were eliminated. This yielded a total of 36 papers. Four of this group of 36 papers, all relating to natural background radiation exposure, were deemed uninformative for radiation risk, but are nevertheless given in Supplementary Table S6.

An additional 24 papers were found by screening recent review papers and other material as indicated above. Of the total of 49 papers that are cited in Tables 1-5, 32 were derived from the PubMed search, while of the 11 papers cited in Supplementary Table S6 that did not overlap with these 49, 4 were derived from the PubMed search, respectively. A number of papers, for example relating to the high natural background radiation studies (Nair et al. 2009; Tao et al. 2000) and the Techa River cohort (Krestinina et al. 2013), did not provide practicable data on childhood exposures as it was not possible to separate the effects of exposure in childhood and in adulthood; as such they were deemed inapplicable. The study of Spix *et al* (Spix et al. 2017) could not be used because the dose-rate metric was not consistent with the cumulative dose measure we generally employ. A few studies were omitted because of known methodological problems or because the data presented was of a preliminary nature (Court Brown et al. 1960; Hirayama 1979; Robinette and Jablon 1976). Some studies of Chernobyl-exposed populations were also deemed inapplicable because they were neither low/moderate dose nor low/moderate dose rate. For example, in the thyroid screening studies in Ukraine and Belarus the maximum cumulative thyroid dose exceeded 30 Gy, with maximum dose rates in excess of 0.12 Gy/hour (Little et al. 2014; Little et al. 2015).

Peak individual dose rate (*DR*) in all the studies of Chernobyl thyroid cancer were derived from individual cumulative dose (*D*) via the relation $DR=D\ln[2]/HL$, where $HL\approx193.4\text{ hours}$is the physical half life of ^131^I, derived from integration of the exponential decay relationship, and this was used mean individual peak and maximum dose rates; this calculation assumes a single discrete intake of ^131^I, which is thought to be approximately true. A similar calculation was done in relation to a study in which ^59^Fe was administered to pregnant women (Hagstrom et al. 1969), making use of the physical half life $HL\approx1068\text{ hours}$ of ^59^Fe.

In the studies of Delongchamp *et al* (Delongchamp et al. 1997), Lundell *et al* (Lundell et al. 1999), Preston *et al* (Preston et al. 2008), Hatch *et al* (Hatch et al. 2019), and Sugiyama *et al* (Sugiyama et al. 2021) data relating to persons exposed <1 Gy in the published material were refitted in order to yield information on moderate dose risk. Three studies of childhood cardiac catheterisation were not included because of lack of individual doses (McLaughlin et al. 1993; Modan et al. 2000; Spengler et al. 1983); the later study of Harbron *et al* (Harbron et al. 2018) was also not used because of lack of sufficient detail on the disease endpoint being used. However, the catheterisation study of Stern *et al* (Stern et al. 2020) was employed.

There is considerable overlap of the leukaemia cases in Ukraine in the studies of Davis *et al* (Davis et al. 2006) and Noshchenko *et al* (Noshchenko et al. 2010). Davis *et al* (Davis et al. 2006) employ *in utero* dose in their risk evaluations whereas Noshchenko *et al* (Noshchenko et al. 2010) focus on leukaemia in relation to postnatal dose, and so the latter has a slightly more straightforward interpretation; for this reason in all analysis we use the Belarus and Russian parts of the data of Davis *et al* (Davis et al. 2006), in conjunction with the Ukraine data of Noshchenko *et al* (Noshchenko et al. 2010). There are grounds for thinking that risks of thyroid nodule and thyroid cancer are not dissimilar, as for example suggested by the Ukraine *in utero* data of Hatch *et al* (Hatch et al. 2019), likewise among postnatally exposed groups in Belarus (Cahoon et al. 2017a; Zablotska et al. 2011). For this reason we group thyroid cancer and thyroid nodules together in all meta-analyses (Tables 6, 7, Supplementary Tables S3-S5).

*S.2 Meta-analysis*

Meta-analysis was conducted of the studies in which there was information on radiation dose, outlined in Tables 1-5; the basis of all estimations of radiation risk in this latter analysis is the value of ERR per unit of absorbed dose of radiation exposure (ERR per Gy). For absorbed dose, most publications employed unweighted radiation dose (Gy), but some use weighted dose, for example in the LSS to account for the higher biological effectiveness of neutrons compared with photons (Shimizu et al. 2010; Grant et al. 2017). Wherever possible the OR, RR or ERR were taken directly from the relevant publication; further details are given in Tables 1-5. Data abstraction from the published papers was performed independently by MPL, GMK and NH. Table S1 gives details of how a few ERR or OR were derived from the published material, when these were not given directly; data abstraction and statistical analysis for these studies was performed independently by MPL and KA. Supplementary Table S2 details the particular studies used in the meta-analyses.

We explored sensitivity analyses in which we refitted thyroid nodule data <0.799 Gy of Hatch *et al* (Hatch et al. 2019), thyroid cancer data <0.284 Gy of Kopecky *et al* (Kopecky et al. 2006), Cardis *et al* (Cardis et al. 2005) thyroid cancer data using a linear model restricted to <1 Gy, Lubin *et al* (Lubin et al. 2017) data restricted to <0.1 Gy, Preston *et al* (Preston et al. 2007) brain/CNS and breast cancer data restricted to <0.1 Gy, Cahoon *et al* (Cahoon et al. 2017b) lung cancer data restricted to <0.1 Gy; these we term the “lower dose risks”. These we contrasted with using full dose range thyroid nodule data of Hatch *et al* (Hatch et al. 2019), the thyroid cancer data of Kopecky *et al* (Kopecky et al. 2006), Cardis *et al* (Cardis et al. 2005) thyroid cancer data using a linear model restricted to <2 Gy, Lubin *et al* (Lubin et al. 2017) data restricted to <0.2 Gy, Preston *et al* (Preston et al. 2007) brain/CNS and breast cancer data restricted to <1 Gy and Cahoon *et al* (Cahoon et al. 2017b) lung cancer data restricted to <1 Gy; these we term the “higher dose risks”.

The principal random effects meta-analysis given in Tables 6 and 7 used estimates derived from a binomial odds model to refit thyroid nodule data <0.799 Gy of Hatch *et al* (Hatch et al. 2019) and leukaemia data of Stevens *et al* (Stevens et al. 1990), and an inverse-variance weighted linear model to refit thyroid cancer data <0.284 Gy of Kopecky *et al* (Kopecky et al. 2006). The reason for this was that the crude OR in the study of Kopecky *et al* (Kopecky et al. 2006) were much less than the adjusted estimates given in the paper, unlike the other two studies. However, in Supplementary Tables S3 and S5 we also provide a similar meta-analysis in which an inverse-variance weighted linear model was used to derive EOR in all three studies. There is some evidence that the ERR following exposure *in utero* and in early life strongly varies with attained age, as documented by Preston *et al* (Preston et al. 2008). We attempted to take this into account in the analyses of Supplementary Tables S4 and S7 by applying adjustments proportional to [mean attained age]^-1.3^ to adjust all solid cancer (and thyroid nodule) ERR to the mean attained age of 6.37 years in the study of Bithell and Stiller (Bithell and Stiller 1988) and Bithell (Bithell 1993). For haemopoietic malignancies a very similar adjustment was applied, [mean attained age]^-1.309^, derived via fit of a stratified linear-quadratic ERR model to all radiogenic leukaemias (acute lymphocytic leukaemia, acute myeloid leukaemia, chronic myeloid leukaemia) exposed under the age of 20, with adjustment for ln[attained age], using strata of city, sex, age at exposure, age and calendar time in data of Hsu *et al* (Hsu et al. 2013), omitting persons not in either city (Hiroshima, Nagasaki) at the time of the bombings, or those with shielded kerma dose ≥ 4 Gy.

An aggregate estimate of ERR per Gy was computed across subsets of these studies using random effects models, using standard statistical methods (Viechtbauer 2010). Random effects models were fitted by restricted maximum likelihood (REML) because of the theoretically superior performance, in particular the absence of bias in the estimate of variance (Bartlett and Fowler 1937; Viechtbauer 2005), and the associated results are given in Table 6 and Supplementary Tables S3 and S4. The details of the data used for these analyses is given in Supplementary Table S2 (relating to Tables 1-5). To facilitate REML and maximum-likelihood algorithm convergence, we limited analyses to data with variance <10^10^, which resulted in loss of five records; despite this problems of convergence persisted for certain model fits, and in these cases we employed instead of the REML model the 1-step random effects model of DerSimonian and Laird (DerSimonian and Laird 1986). For certain analyses (Supplementary Table S6) maximum-likelihood fits were used, as these facilitate comparison of nested models, in particular tests of improvement in fit relative to the null. Residual heterogeneity was assessed using Cochran’s Q-statistic, the significance of which was assessed by comparing it against centiles of the $\chi^{2}$ distribution with the relevant number of degrees of freedom ($=N-1$). The 2-sided *p*-values in Tables 6, 7, Supplementary Tables S3-S5 were calculated in the standard way. Statistical significance was defined by *p*<0.05. In order to assess the contribution of the heterogeneity to the aggregate data the *I*^2^ statistic of Higgins and Thompson (Higgins and Thompson 2002) is computed. This is expressed as a percentage, so that a value near 0% implies little estimated inter-study heterogeneity relative to the intra-study variance, and values near 100% that the inter-study heterogeneity dominates the intra-study variance (Higgins and Thompson 2002). Values of ERR per Gy derived from the meta-analysis are given in Table 6 and 7 for major cancer subtypes (leukaemia, lymphoma, brain/CNS etc), by *in utero* vs postnatal exposure and by study type.

In order to assess selection or publication bias, funnel plots are employed. Funnel plots are scatterplots of the central estimate of risk against estimates of standard error, and as discussed by Egger *et al* (Egger et al. 1997; Sterne and Egger 2001) are useful qualitative means of detecting various types of selection bias, in particular publication bias. If the funnel plot has the form of an inverted symmetric funnel then selection bias is thought to be unlikely (Egger et al. 1997; Sterne and Egger 2001). More formal tests of selection or publication bias are also conducted using the test statistic suggested by Egger *et al* (Egger et al. 1997). We also employ the trim-and-fill method of Duval and Tweedie (Duval and Tweedie 2000) to assess the likely magnitude of the change in ERR that may result from selection bias. All statistical models, including funnel plots, are fitted using the metafor package (Viechtbauer 2010; 2020) in R (R Project version 3.6.1 2019).

**Supplementary Table S1. Specification of data extraction for various studies in which excess relative risk (ERR) or odds ratio (OR) were not given directly**

| Reference | Description of study data | Details of data extraction | Model fitting notes | Endpoint (incidence unless otherwise stated) |
| --- | --- | --- | --- | --- |
| **Table 2** | | | | |
| (Lundell et al. 1999) | Swedish haemangioma cohort irradiated for skin haemangioma at Radiumhemmet Stockholm 1920-1959 and Sahlgrenska University Hospital Göteborg 1930-1965 and followed up 1958-1993 | Use mean breast dose, breast years of follow-up and numbers of cases from Table 4 | Refitted using Poisson linear model restricted to <1 Gy data | Breast |
| (Hagstrom et al. 1969) | Women administered ^59^Fe in pregnancy at Vanderbilt University Hospital, 1945-1949 and followed up to 1967 | Construct 2 x 2 tables for numbers of exposed and unexposed for each endpoint and the respective controls | Derive odds ratio (OR) estimated via maximum likelihood from hypergeometric model conditional on marginal totals, with exact CI, estimated by fisher.test routine in R (R Project version 3.6.1 2019), divide excess OR (= OR - 1) by mean dose (0.1 Gy) | Leukaemia mortality |
| (Pasqual et al. 2020) | MOBI-Kids multinational case-control study of medical diagnostic radiation exposures among persons aged 10-24 y, OR of >5 mGy vs < 5 mGy | The maximum dose is 0.0127 Gy, so the mean difference in dose between the <5 mGy and >5 mGy groups is less than this. As the mean dose overall is <0.00073 Gy (Table 2), so must the mean in the <5 mGy group also be less than this, so the mean dose difference (<5 mGy vs >5 mGy) must be >0.00427 Gy and <0.0127 Gy. It is a reasonable guess, given the highly skewed nature of the dose distribution (Figure 1), that it would not be much more than 0.00427 Gy. | ERR derived by dividing RR - 1 for >5 mGy vs 0-5 mGy (Table 3) by the presumed mean dose difference, ~0.005 Gy | Brain/CNS |
| **Table 3** | | | | |
| (Hatch et al. 2019) | Ukraine in utero ^131^I exposed cohort | Use numbers of thyroid nodules, no nodules and mean dose by dose groups, from Table 2 | Refitted using a linear binomial odds model, with the denominator = nodules + no nodules, using mean dose as regressor, using data < 0.799 Gy | Thyroid nodule |
|  |  | Excess odds ratio and 95% CI [CI_l_, CI_u_] and mean doses from Table 2. Derive variance for each dose group via [[CI_u_ - CI_l_] / (2*1.96)]^2^ | Analysis of EOR in relation to mean dose restricted to < 0.799 Gy, refitted by inverse variance-weighted linear model to data, using mean dose as regressor |  |
| (Johnson et al. 2008) | US Radiologic Technologists offspring born 1921-1984 | Use numbers of malignancies and person years from Table 3, and use midpoints of dose ranges as estimates of mean dose | Adjusted for birth year, refitted from published data via Poisson linear model | Leukaemia |
|  |  |  |  | Lymphoma |
|  |  |  |  | Solid cancer |
|  |  |  |  | All cancer |
| (Bunch et al. 2009) | Offspring of female members of UK National Registry for Radiation Workers | Construct 2 x *n* tables for numbers of eases and controls by dose group from Table 1 for each endpoint | Linear binomial odds model fitted to data from paper with the denominator = cases + controls and using midpoint dose (0.003 Gy for >0.002 Gy) as regressor. | Leukaemia and NHL |
|  |  |  |  | Cancer other than leukaemia and NHL |
| **Table 4** | | | | |
| (Preston et al. 2007) | LSS brain/CNS cancer incidence 1958-1998, DS02 brain dose | Use freely downloadable data from RERF website | Refitted to downloadable data <1 Gy brain dose, age at exposure < 20 y, via Poisson linear model, stratified by age at exposure, age, sex, city, distance category | Brain/CNS |
|  |  |  | Refitted to downloadable data <0.2 Gy brain dose, age at exposure < 20 y, via Poisson linear model, stratified by age at exposure, age, sex, city, distance category |  |
|  |  |  | Refitted to downloadable data <0.1 Gy brain dose, age at exposure < 20 y, via Poisson linear model, stratified by age at exposure, age, sex, city, distance category |  |
|  | LSS breast cancer incidence 1958-1998, DS02 breast dose | Use freely downloadable data from RERF website | Refitted to downloadable data <1 Gy breast dose, age at exposure < 20 y, via Poisson linear model, stratified by age at exposure, age, sex, city, distance category | Breast |
|  |  |  | Refitted to downloadable data <0.2 Gy breast dose, age at exposure < 20 y, via Poisson linear model, stratified by age at exposure, age, sex, city, distance category |  |
|  |  |  | Refitted to downloadable data <0.1 Gy breast dose, age at exposure < 20 y, via Poisson linear model, stratified by age at exposure, age, sex, city, distance category |  |
| (Cahoon et al. 2017b) | LSS incidence 1958-2009, DS02R1 dose | Use freely downloadable data from RERF website | Refitted to downloadable data <1 Gy lung dose, age at exposure < 20 y, via Poisson linear model, stratified by age at exposure, age, sex, city, distance category | Lung |
|  |  |  | Refitted to downloadable data <0.2 Gy lung dose, age at exposure < 20 y, via Poisson linear model, stratified by age at exposure, age, sex, city, distance category |  |
|  |  |  | Refitted to downloadable data <0.1 Gy lung dose, age at exposure < 20 y, via Poisson linear model, stratified by age at exposure, age, sex, city, distance category |  |
| (Grant et al. 2017) | LSS incidence 1958-2009, DS02R1 dose | Use freely downloadable data from RERF website | Refitted to downloadable data <1 Gy colon dose, age at exposure < 20 y, via Poisson linear model, stratified by age at exposure, age, sex, city, distance category | Solid |
|  |  |  | Refitted to downloadable data <0.2 Gy colon dose, age at exposure < 20 y, via Poisson linear model, stratified by age at exposure, age, sex, city, distance category |  |
|  |  |  | Refitted to downloadable data <0.1 Gy colon dose, age at exposure < 20 y, via Poisson linear model, stratified by age at exposure, age, sex, city, distance category |  |
| (Delongchamp et al. 1997) | LSS in utero mortality with DS86 doses, followed up to age 47 or 1997 | Use leukaemia and solid cancer deaths after in utero exposure, and product of number of persons and average PY of follow-up (to yield person year), with midpoint of each interval of dose as the mean, and stratified by sex, all from Table VI | Restricted to maternal uterine dose < 1 Gy, refitted via Poisson linear model stratified by sex, using published data | Leukaemia mortality |
|  |  |  |  | Solid cancer mortality |
| (Preston et al. 2008) | LSS in utero cohort, DS02 doses, followed to 1999 or age 55 | Use solid cancer cases after in utero exposure, and number of person years of follow-up, with midpoint of each interval of dose as the mean, all from Table 3 | Refitted to published data maternal uterine dose < 1 Gy via Poisson linear model | Solid cancer |
| (Sugiyama et al. 2021) | LSS in utero mortality with DS02R1 doses, followed up 1950-2012, maternal uterine dose | Use cancer deaths by endpoint and dose group, and number of person years of follow-up, with midpoint of each interval of dose as the mean (but the 0.1-1 Gy via person year weighted estimates from Table 3 of (Preston et al. 2008)), all from Table 3 | Refitted to published data maternal uterine dose < 1 Gy via Poisson linear relative risk model, stratified by sex | Solid cancer mortality |
|  |  |  |  | Esophageal cancer mortality |
|  |  |  |  | Stomach cancer mortality |
|  |  |  |  | Colon cancer mortality |
|  |  |  |  | Rectal cancer mortality |
|  |  |  |  | Liver cancer mortality |
|  |  |  |  | Pancreas cancer mortality |
|  |  |  |  | Lung cancer mortality |
|  |  |  |  | Lymphohaemopoietic mortality |
|  |  |  |  | Leukaemia mortality |
| **Table 5** | | | | |
| (Stevens et al. 1990) | Utah fallout case-control study 1952-1981 | Numbers of cases and controls, with midpoint doses per dose group as mean, all from Table 1 | Linear binomial odds model fitted to data from paper with the denominator = cases + controls and using midpoint dose as regressor. | Leukaemia |
|  |  |  |  | Chronic lymphocytic leukaemia |
|  |  |  |  | Leukaemia excluding CLL |
|  |  | Odds ratios (OR) and 95% CI [CI_l_, CI_u_], with dose intervals from Table 1. Derive variance for each dose group via [[CI_u_ - CI_l_] / (2*1.96)]^2^ | Analysis refitted by inverse variance-weighted linear model to OR data and using midpoint dose as regressor |  |
| (Kopecky et al. 2006) | Bryansk case-control study 1986-1998, persons aged < 20 y at time of Chernobyl accident | Median doses, numbers of cases and controls from Table 2. | Analysis restricted to <0.284 Gy, linear binomial odds model refitted to data from paper with the denominator = cases + controls and using median dose as regressor. | Thyroid |
|  |  | Odds ratio (OR) and 95% CI [CI_l_, CI_u_] and median doses from Table 2. Derive variance for each dose group via [[CI_u_ - CI_l_] / (2*1.96)]^2^ | Analysis restricted to <0.284 Gy, refitted by inverse variance-weighted linear model to OR data and using median dose as regressor |  |
| (Ohira et al. 2020) | Fukushima cohort study 2011-2017, ages 6-14, aged < 18 y at time of Fukushima accident | Relative risks (RR) and 95% CI [CI_l_, CI_u_], with dose intervals from Table 2. Derive variance for each dose group via [[CI_u_ - CI_l_] / (2*1.96)]^2^ | Inverse-variance weighted linear model refitted to RR data by dose group from paper and using midpoint dose as regressor | Thyroid |

**Supplementary Table S2. Studies used in meta analysis of Tables 6 and 7 and Supplementary Tables S3, S4 (using data taken from Tables 1-5)**

| Reference | Endpoint [incidence unless otherwise noted] | Description | Notes | *In utero* / post natal | Mapped endpoint in Tables 6+7 |
| --- | --- | --- | --- | --- | --- |
| **Table 1** | | | | |  |
| (Kendall et al. 2013) | Leukaemia | Great Britain NRCT study 1980-2006 | Gamma only, mean equivalent RBM dose and range including dose from radon and gamma | Postnatal | Leukaemia |
|  | Brain/CNS |  |  | Postnatal | Brain/CNS |
|  | Lymphoma |  |  | Postnatal | Lymphoma (including CLL) |
| (Spycher et al. 2015) | Leukaemia | Swiss Cancer Registry study, 1990-2008, children < 16 y | Gamma only, mean equivalent RBM dose and range including dose from radon and gamma | Postnatal | Leukaemia |
|  | Brain/CNS |  |  | Postnatal | Brain/CNS |
|  | Lymphoma |  |  | Postnatal | Lymphoma (including CLL) |
| (Nikkilä et al. 2016) | Leukaemia | Finnish Cancer Registry study 1990-2011 | median doses, dose rates in controls | Postnatal | Leukaemia |
| (Demoury et al. 2017) | Acute leukaemia | French Childhood Cancer Registry (RNCE) ecological study 1990-2009, and case-control study 2002-2007 | mean, max dose and mean, max dose rate estimated from controls age 15 in Geocap case-control study | Postnatal | Leukaemia |
| (Berlivet et al. 2020) | Brain/CNS | French Childhood Cancer Registry (RNCE) ecological study 2000-2012 |  | Postnatal | Brain/CNS |
| **Table 2** | | | | |  |
| (Pottern et al. 1990) | Thyroid nodule | Lymphoid hyperplasia cohort treated 1938-1969 at Children's Hospital Medical Center, Boston | Examination data, mean dose weighted sum of exposed and unexposed | Postnatal | Thyroid |
| (Little and Boice 1999) | Breast | Massachusetts TB fluoroscopy cohort, with exposure under age 20 | Adjustment for attained age (centred at age 50) | Postnatal | All solid except brain/CNS, lung, thyroid |
| (Lundell et al. 1999) | Breast | Swedish haemangioma cohort irradiated for skin haemangioma at Radiumhemmet Stockholm 1920-1959 and Sahlgrenska University Hospital Göteborg 1930-1965 and followed up 1958-1993 | Refitted to data in paper <1 Gy using a linear Poisson model, mean dose via breast year weighted mean, maximum dose rate from Lundell (Lundell 1994) | Postnatal | All solid except brain/CNS, lung, thyroid |
| (Ronckers et al. 2010) | Breast mortality | US scoliosis cohort of women diagnosed with scoliosis 1912-1965 and followed (via linkage with various registers) up to 2004, given multiple diagnostic X-radiographs | Mean and maximum dose rate based on the mean and maximum breast dose per radiograph, from Ronckers *et al* (Ronckers et al. 2008) | Postnatal | All solid except brain/CNS, lung, thyroid |
|  | Lung mortality | US scoliosis cohort of women diagnosed with scoliosis 1912-1965 and followed (via linkage with various registers) from 1992 (date of questionnaire) to 2004, given multiple diagnostic X-radiographs | Mean and maximum dose rate based on the mean and maximum breast dose per radiograph, from Ronckers *et al* (Ronckers et al. 2008), scaled by ratio between mean lung dose and mean breast dose | Postnatal | Lung |
| (Pearce et al. 2012) | Brain | UK-NCI paediatric CT cohort, age at first CT < 21 y, 1985-2008 |  | Postnatal | Brain/CNS |
| (Mathews et al. 2013) | Leukaemia + MDS | Australian CT study of persons aged 0-19 years in 1/1985 or born after that point, 1985-2007 | 1 year lag, dose rates based on mean dose per scan | Postnatal | Leukaemia |
|  | Brain/CNS |  | 5 year lag, dose rates based on mean dose per scan | Postnatal | Brain/CNS |
| (Journy et al. 2015) | Leukaemia | French infant CT study, 2000-2011, age < 10 y at first CT, followed via RNCE | Median RBM dose - very similar to Journy *et al* (Journy et al. 2016) except this paper uses Poisson models with linear ERR model. 2 year exclusion | Postnatal | Leukaemia |
|  | Brain/CNS |  | Median brain dose - very similar to Journy *et al* (Journy et al. 2016) except this paper uses Poisson models with linear ERR model. 4 year exclusion | Postnatal | Brain/CNS |
|  | Lymphoma |  | Median RBM dose - very similar to Journy *et al* (Journy et al. 2016) except this paper uses Poisson models with linear ERR model. 2 year exclusion | Postnatal | Lymphoma (including CLL) |
| (Krille et al. 2015) | Leukaemia | German infant CT study, 1980-2010, age < 15 y at first CT, lag 2 y | Using ABM dose | Postnatal | Leukaemia |
|  | Brain/CNS |  | Using brain dose | Postnatal | Brain/CNS |
| (Berrington de Gonzalez et al. 2017) | Hodgkin lymphoma | UK-NCI paediatric CT cohort, age at first CT < 22 y, 1980-2008 | Using RBM dose, 2 y lag | Postnatal | Lymphoma (including CLL) |
| (Lubin et al. 2017) | Thyroid | Pooled analysis of 9 datasets, dose < 0.2 Gy |  | Postnatal | Thyroid |
|  | Thyroid | Pooled analysis of 9 datasets, dose < 0.1 Gy |  | Postnatal | Thyroid |
| (Little et al. 2018b) | Acute myeloid leukaemia + MDS | 9 cohort pooled moderate dose medical+LSS analysis - dose < 0.1 Gy |  | Postnatal | Leukaemia |
|  | Acute lymphocytic leukaemia |  |  | Postnatal | Leukaemia |
|  | Chronic myeloid leukaemia |  |  | Postnatal | Leukaemia |
| (Little et al. 2018a) | Thyroid | US Radiologic Technologist cohort, followed up via four questionnaires administered 1983-2014 | Health endpoints and medical diagnostic exposure assessed via questionnaire. Risks for exposure aged 0-19 as part of model with separate windows for various age at exposure groups | Postnatal | Thyroid |
| (Nikkila et al. 2018) | Leukaemia | Finnish Cancer Registry based case-control study 1990-2011 | median dose for controls, using NCICT software | Postnatal | Leukaemia |
| (Meulepas et al. 2019) | Brain/CNS | Dutch CT study of children (age < 18 y) at first CT, 1979-2012 | Exclusion and lag 5 y | Postnatal | Brain/CNS |
|  | Leukaemia + MDS |  | Exclusion and lag 2 y | Postnatal | Leukaemia |
| (Kojimahara et al. 2020) | Brain/CNS | Case-control study of 120 cases and 360 appendicitis controls aged 10-24 y, 2011-2015 | Mean dose to controls, lag 2 y, adjusted for parental education, history of neurological disease and ADD/ADHD | Postnatal | Brain/CNS |
| (Pasqual et al. 2020) | Brain/CNS | MOBI-Kids multinational case-control study of medical diagnostic radiation exposures among persons aged 10-24 y | Dose lag 2 y | Postnatal | Brain/CNS |
| (Zidane et al. 2021) | Thyroid | French CATHY three-centre case-control study, with focus on childhood medical diagnostic procedures 2002-2006 | Linear part of linear-quadratic-exponential dose response | Postnatal | Thyroid |
| (Hagstrom et al. 1969) | Leukaemia mortality | Women administered ^59^Fe in pregnancy at Vanderbilt University Hospital, 1945-1949 and followed up to 1967 | Dose rate estimated via theoretical calculation based on physical half life of ^59^Fe, excess relative risk derived via dividing excess odds ratio by mean dose | *In utero* | Leukaemia |
|  | Lymphoma mortality |  |  | *In utero* | Lymphoma (including CLL) |
|  | Solid tumour mortality |  |  | *In utero* | All endpoints |
| (Bithell 1993) | All cancer mortality | Oxford Survey of Childhood Cancers, case-control pairs born 1953-1979 | Estimated via log linear-quadratic model of OR fitted to data of Gilman *et al* (Gilman et al. 1988) by year for 1959, using dose estimate of 6.1 mGy per obstetric radiograph of Mole (Mole 1990) for that year | *In utero* | All endpoints |
| (Pasqual et al. 2020) | Brain/CNS | MOBI-Kids multinational case-control study of medical diagnostic radiation exposures among persons aged 10-24 y, OR of >5 mGy vs < 5 mGy | Dose lag 2 y, ERR derived by dividing ERR for >5 mGy vs 0-5 mGy by 0.005 (the mean dose difference between >5 mGy and <5 mGy groups must be between 0.00427 and 0.0127 Gy, and assumed to be ~0.005 Gy given the skewed nature of the dose distribution) | *In utero* | Brain/CNS |
| **Table 3** | | | | |  |
| (Akleyev et al. 2016) | Solid cancer | Techa River and Mayak Worker cohorts 1950-2009 |  | *In utero* | All endpoints |
| (Schüz et al. 2017) | Haematological malignancy | Techa River and Mayak workers exposed/followed-up 1953-2009 (TR incidence), 1950-2009 (TR mortality), 1948-2009 (MW) | median (among those without malignancy) rather than mean dose | *In utero* | All endpoints |
| (Hatch et al. 2019) | Thyroid | Ukraine in utero ^131^I exposed cohort |  | *In utero* | Thyroid |
|  | Thyroid nodule |  |  | *In utero* | Thyroid |
|  | Thyroid nodule |  | Refitted using binomial odds model using data from paper < 0.799 Gy | *In utero* | Thyroid |
|  | Thyroid nodule |  | Refitted using inverse-variance reweighted least squares model using data from paper < 0.799 Gy | *In utero* | Thyroid |
| (Johnson et al. 2008) | Leukaemia | US Radiologic Technologists offspring born 1921-1984 | Refitted from published data via Poisson linear model | *In utero* | Leukaemia |
|  | Lymphoma |  | Refitted from published data via Poisson linear model | *In utero* | Lymphoma |
|  | All cancer |  | Refitted from published data via Poisson linear model | *In utero* | All endpoints |
| (Bunch et al. 2009) | Leukaemia and NHL | Offspring of female members of UK National Registry for Radiation Workers | Refitted from published data via linear binomial odds model | *In utero* | All endpoints |
|  | Cancers others than leukaemia and NHL |  | Refitted from published data via linear binomial odds model | *In utero* | All endpoints |
| **Table 4** | | | | |  |
| (Preston et al. 2007) | Brain/CNS | LSS brain/CNS cancer incidence 1958-1998, DS02 dose | Refitted to downloadable data <1 Gy brain dose, age < 20 y at exposure, via Poisson linear model, stratified by age at exposure, age, sex, city, distance category | Postnatal | Brain/CNS |
|  | Brain/CNS |  | Refitted to downloadable data <0.1 Gy brain dose, age < 20 y at exposure, via Poisson linear model, stratified by age at exposure, age, sex, city, distance category | Postnatal | Brain/CNS |
|  | Breast | LSS incidence 1958-1998, DS02 dose | Refitted to downloadable data <1 Gy breast dose, age < 20 y at exposure, via Poisson linear model, stratified by age at exposure, age, sex, city, distance category | Postnatal | All solid except brain/CNS, lung, thyroid |
|  | Breast |  | Refitted to downloadable data <0.1 Gy breast dose, age < 20 y at exposure, via Poisson linear model, stratified by age at exposure, age, sex, city, distance category | Postnatal | All solid except brain/CNS, lung, thyroid |
| (Cahoon et al. 2017b) | Lung | LSS incidence 1958-2009, DS02R1 dose | Refitted to downloadable data <1 Gy lung dose, age < 20 y at exposure, via Poisson linear model, stratified by age at exposure, age, sex, city, distance category | Postnatal | Lung |
|  | Lung |  | Refitted to downloadable data <0.1 Gy lung dose, age < 20 y at exposure, via Poisson linear model, stratified by age at exposure, age, sex, city, distance category | Postnatal | Lung |
| (Delongchamp et al. 1997) | Leukaemia mortality | LSS *in utero* mortality with DS86 doses, followed up to age 47 or 1997 | Restricted to maternal uterine dose < 1 Gy, refitted via Poisson linear model stratified by sex, using published data | *In utero* | Leukaemia |
| (Preston et al. 2008) | Solid | LSS *in utero* cohort, DS02 doses, followed to 1999 or age 55 | Refitted to published data maternal uterine dose < 1 Gy via Poisson linear relative risk model | *In utero* | All endpoints |
| (Sugiyama et al. 2021) | Esophageal cancer mortality | LSS *in utero* mortality with DS02R1 doses, followed up 1950-2012, maternal uterine dose | Refitted to published data maternal uterine dose < 1 Gy via Poisson linear relative risk model, stratified by sex | *In utero* | All solid except brain/CNS, lung, thyroid |
|  | Stomach cancer mortality |  |  | *In utero* | All solid except brain/CNS, lung, thyroid |
|  | Colon cancer mortality |  |  | *In utero* | All solid except brain/CNS, lung, thyroid |
|  | Rectal cancer mortality |  |  | *In utero* | All solid except brain/CNS, lung, thyroid |
|  | Liver cancer mortality |  |  | *In utero* | All solid except brain/CNS, lung, thyroid |
|  | Pancreas cancer mortality |  |  | *In utero* | All solid except brain/CNS, lung, thyroid |
|  | Lung cancer mortality |  |  | *In utero* | Lung |
|  | Leukaemia mortality |  |  | *In utero* | Leukaemia |
| **Table 5** | | | | |  |
| (Stevens et al. 1990) | Chronic lymphocytic leukaemia | Utah fallout case-control study 1952-1981 | Inverse-variance weighted linear model fitted to data from paper. Median dose for all cases and controls used as mean | Postnatal | Lymphoma (including CLL) |
|  | Leukaemia excluding CLL | Utah fallout case-control study 1952-1981 | Inverse-variance weighted linear model fitted to data from paper. Median dose for all cases and controls used as mean | Postnatal | Leukaemia |
| (Parkin et al. 1996) | Leukaemia | Ecologic analysis of cancer in 23 European countries after Chernobyl nuclear accident 1980-1991 | ERR/Gy derived via ERR/Sv ± 1.96 SE. Mean dose derived via case-weighted average, using midpoint dose estimates | Postnatal | Leukaemia |
| (Davis et al. 2004) | Thyroid | Hanford cohort study of sampled births 1940-1946 in eastern Washington State, followed to 1997 |  | Postnatal | Thyroid |
|  | Benign thyroid nodules |  |  | Postnatal | Thyroid |
| (Cardis et al. 2005) | Thyroid | Belarus & Russia thyroid cancer case-control study, 1992-1998, after ^131^I exposure from Chernobyl, persons aged < 15 y at time of accident | Linear model <1 Gy, OR at 1 Gy | Postnatal | Thyroid |
|  | Thyroid |  | Linear model <2 Gy, OR at 1 Gy | Postnatal | Thyroid |
| (Davis et al. 2006) | Leukaemia | Belarus part of three country (Belarus, Russia and Ukraine) case-control study 1986-2000, children aged < 6 y or *in utero* at time of Chernobyl accident | Mean dose among controls | Postnatal | Leukaemia |
|  | Leukaemia | Russian part of three country (Belarus, Russia and Ukraine) case-control study 1986-2000, children aged < 6 y or *in utero* at time of Chernobyl accident | Mean dose among controls, using log-linear model | Postnatal | Leukaemia |
| (Kopecky et al. 2006) | Thyroid | Bryansk case-control study 1986-1998, persons aged < 20 y at time of Chernobyl accident | Median dose for controls used as mean | Postnatal | Thyroid |
|  | Thyroid |  | Median dose in middle dose group used for mean, analysis restricted to <0.284 Gy, refitted by linear binomial odds model to data from paper | Postnatal | Thyroid |
|  | Thyroid |  | Median dose in middle dose group used for mean, analysis restricted to <0.284 Gy, refitted by inverse-variance weighted linear model to data from paper | Postnatal | Thyroid |
| (Lyon et al. 2006) | Thyroid | Utah, Nevada, Arizona fallout cohort study 1965-1998 among persons aged 12-18 y in 1965-1966, Phase IIR analysis |  | Postnatal | Thyroid |
|  | Thyroid neoplasms |  |  | Postnatal | Thyroid |
|  | Thyroid nodule |  |  | Postnatal | Thyroid |
| (Noshchenko et al. 2010) | Leukaemia | Study cases among children (age 0-5) of four most highly Chernobyl-contaminated regions (oblasts) of Ukraine diagnosed 1987-1997 | Mean dose in controls, linear part of dose response used for regression coefficient | Postnatal | Leukaemia |
| (Ohira et al. 2020) | Thyroid | Fukushima cohort study 2011-2017, ages 6-14, aged < 18 y at time of Fukushima accident | Inverse-variance weighted linear model refitted to RR data, adjusted for age, sex, examination year, by dose group from paper. Mean dose estimated via midpoint of each dose group weighted by persons | Postnatal | Thyroid |

**Supplementary Table S3. Alternative restricted maximum likelihood (REML) and DerSimonian and Laird 1-step random effects model fits to various subsets (as given by Supplementary Table S2)**^a^

| Endpoint | Excess relative risk (ERR) / Gy (95% CI) | *p*-value | Residual heterogeneity *p*-value | *I*^2^ (%) |
| --- | --- | --- | --- | --- |
| Analysis using lower dose risk estimates^b^ | | | | |
| Postnatal exposure | | | | |
| Leukaemia | 1.31 (0.96,1.66) | <0.001 | 0.036 | 0.00 |
| Lymphoma (including CLL) | 0.45 (-12.83,13.74) | 0.947 | 0.981 | 0.00 |
| Brain/CNS | 6.81 (0.58,13.04) | 0.032 | <0.001 | 78.57 |
| Lung | 2.23 (-5.55,10.02) | 0.574 | 0.069 | 69.87 |
| Thyroid (including nodules) | 0.00 (-0.07,0.08)^c^ | 0.928^c^ | <0.001^c^ | 65.64^c^ |
| All solid except brain/CNS, lung, thyroid | 0.00 (-0.41,0.42) | 0.983 | 0.189 | 0.00 |
| All six endpoints only^d^ | 0.23 (0.07,0.39)^c^ | 0.006^c^ | <0.001^c^ | 72.31^c^ |
| All endpoints^d^ | 0.26 (0.10,0.42)^c^ | 0.002^c^ | <0.001^c^ | 73.57^c^ |
| *In utero* exposure | | | | |
| Leukaemia | -2.70 (-11.07,5.67) | 0.527 | 0.966 | 0.00 |
| Lymphoma (including CLL) | 229.10 (-200.94,659.14) | 0.296 | 1.000 | 0.00 |
| Brain/CNS | 70.00 (-229.00,369.00) | 0.646 | 1.000 | 0.00 |
| Lung | -1.40 (-3.82,1.03) | 0.258 | 1.000 | 0.00 |
| Thyroid (including nodules) | 2.75 (1.92,3.58) | <0.001 | 0.946 | 0.00 |
| All solid except brain/CNS, lung, thyroid | -1.09 (-2.75,0.56) | 0.196 | 0.754 | 9.23 |
| All six endpoints only^d^ | 0.01 (-1.81,1.83) | 0.989 | <0.001 | 52.46 |
| All endpoints^d^ | 1.13 (-1.00,3.26)^c^ | 0.300^c^ | <0.001^c^ | 73.93^c^ |
| Analysis using higher dose risk estimates^e^ | | | | |
| Postnatal exposure | | | | |
| Leukaemia | -2.70 (-11.07,5.67) | 0.527 | 0.966 | 0.00 |
| Lymphoma (including CLL) | 0.45 (-12.83,13.74) | 0.947 | 0.981 | 0.00 |
| Brain/CNS | 6.87 (1.02,12.72) | 0.021 | <0.001 | 85.15 |
| Lung | 0.41 (-0.23,1.06) | 0.209 | 0.483 | 0.00 |
| Thyroid (including nodules) | 0.01 (-0.08,0.09)^c^ | 0.874^c^ | <0.001^c^ | 73.37^c^ |
| All solid except brain/CNS, lung, thyroid | 0.77 (-0.61,2.14) | 0.274 | 0.007 | 83.57 |
| All six endpoints only^d^ | 0.28 (0.12,0.44)^c^ | <0.001^c^ | <0.001^c^ | 74.59^c^ |
| All endpoints^d^ | 0.41 (0.24,0.58)^c^ | <0.001^c^ | <0.001^c^ | 80.61^c^ |
| *In utero* exposure | | | | |
| Leukaemia | -2.70 (-11.07,5.67) | 0.527 | 0.966 | 0.00 |
| Lymphoma (including CLL) | 229.10 (-200.94,659.14) | 0.296 | 1.000 | 0.00 |
| Brain/CNS | 70.00 (-229.00,369.00) | 0.646 | 1.000 | 0.00 |
| Lung | -1.40 (-3.82,1.03) | 0.258 | 1.000 | 0.00 |
| Thyroid (including nodules) | 1.54 (-0.15,3.22) | 0.074 | 0.890 | 0.00 |
| All solid except brain/CNS, lung, thyroid | -1.09 (-2.75,0.56) | 0.196 | 0.754 | 9.23 |
| All six endpoints only^d^ | -0.38 (-1.87,1.10) | 0.613 | 0.550 | 26.44 |
| All endpoints^d^ | 0.70 (-1.17,2.57)^c^ | 0.463^c^ | 0.006^c^ | 56.70^c^ |

^a^using inverse-variance weighted linear model to refit thyroid nodule data <0.799 Gy of Hatch *et al* (Hatch et al. 2019), leukaemia data of Stevens *et al* (Stevens et al. 1990), and thyroid cancer data <0.284 Gy of Kopecky *et al* (Kopecky et al. 2006)

^b^using refitted thyroid nodule data <0.799 Gy of Hatch *et al* (Hatch et al. 2019), thyroid cancer data <0.284 Gy of Kopecky *et al* (Kopecky et al. 2006), Cardis *et al* (Cardis et al. 2005) thyroid cancer data using a linear model restricted to <1 Gy, Lubin *et al* (Lubin et al. 2017) data restricted to <0.1 Gy, Preston *et al* (Preston et al. 2007) brain/CNS and breast cancer data restricted to <0.1 Gy, Cahoon *et al* (Cahoon et al. 2017b) lung cancer data restricted to <0.1 Gy.

^c^indications of non-convergence for REML model so that 1-step random effects model of DerSimonian and Laird (DerSimonian and Laird 1986) was employed instead.

^d^for “all endpoints” all endpoints were considered, whereas for “all six endpoints” the endpoint considered within a particular study had to lie within one of the six specified endpoints shown.

^e^using full range thyroid nodule data of Hatch *et al* (Hatch et al. 2019), thyroid cancer data of Kopecky *et al* (Kopecky et al. 2006), Cardis *et al* (Cardis et al. 2005) thyroid cancer data using a linear model restricted to <2 Gy, Lubin *et al* (Lubin et al. 2017) data restricted to <0.2 Gy, Preston *et al* (Preston et al. 2007) brain/CNS and breast cancer data restricted to <1 Gy, Cahoon *et al* (Cahoon et al. 2017b) lung cancer data restricted to <1 Gy.

**Supplementary Table S4. Restricted maximum likelihood analysis of studies of *in utero* exposure, analogous to Table 6, with additional adjustment for attained age^a^**

| Endpoint | ERR / Gy (95% CI) | *p*-value | Residual heterogeneity *p*-value | *I*^2^ (%) |
| --- | --- | --- | --- | --- |
| Analysis using lower dose risk estimates^b^ | | | | |
| Leukaemia | -41.15 (-179.46,97.16) | 0.560 | 0.937 | 0.00 |
| Lymphoma (including CLL) | 891.29 (-781.72,2564.31) | 0.296 | 1.000 | 0.00 |
| Brain/CNS | 392.95 (-1285.51,2071.42) | 0.646 | 1.000 | 0.00 |
| Lung | -23.64 (-64.64,17.36) | 0.258 | 1.000 | 0.00 |
| Thyroid (including nodules) | 16.69 (1.33,32.06) | 0.033 | 0.937 | 0.00 |
| All solid except brain/CNS, lung, thyroid | -18.51 (-46.55,9.52) | 0.196 | 0.754 | 9.23 |
| All six endpoints only^c^ | -6.39 (-29.46,16.67) | 0.587 | 0.406 | 29.60 |
| Analysis using higher dose risk estimates^d^ | | | | |
| Leukaemia | -41.15 (-179.46,97.16) | 0.560 | 0.937 | 0.00 |
| Lymphoma (including CLL) | 891.29 (-781.72,2564.31) | 0.296 | 1.000 | 0.00 |
| Brain/CNS | 392.95 (-1285.51,2071.42) | 0.646 | 1.000 | 0.00 |
| Lung | -23.64 (-64.64,17.36) | 0.258 | 1.000 | 0.00 |
| Thyroid (including nodules) | 10.05 (-0.96,21.06) | 0.074 | 0.890 | 0.00 |
| All solid except brain/CNS, lung, thyroid | -18.51 (-46.55,9.52) | 0.196 | 0.754 | 9.23 |
| All six endpoints only^c^ | -7.79 (-28.99,13.42) | 0.472 | 0.519 | 26.79 |

^a^using binomial odds model to refit thyroid nodule data <0.799 Gy of Hatch *et al* (Hatch et al. 2019), and using -1.3 power of attained age to adjust all solid cancer (and thyroid nodule) ERR to mean attained age of 6.37 years in study of Bithell and Stiller (Bithell and Stiller 1988) and Bithell (Bithell 1993), and using [mean attained age]^-1.3^ to adjust all solid cancer (and thyroid nodule) ERR to mean attained age of 6.37 years in study of Bithell and Stiller (Bithell and Stiller 1988), and [mean attained age]^-1.309^ to adjust for all haemopoietic malignancies, derived via fit of stratified linear-quadratic ERR model to all radiogenic leukaemias (acute lymphocytic leukaemia, acute myeloid leukaemia, chronic myeloid leukaemia) exposed under the age of 20, with adjustment for ln[attained age], using strata of city, sex, age at exposure, age and calendar time in data of Hsu *et al* (Hsu et al. 2013), omitting persons not in either city (Hiroshima, Nagasaki) at the time of the bombings, or those with shielded kerma dose ≥ 4 Gy.

^b^using refitted thyroid nodule data <0.799 Gy of Hatch *et al* (Hatch et al. 2019).

^c^the endpoint considered within a particular study had to lie within one of the six specified endpoints shown.

^d^using full range thyroid nodule data of Hatch *et al* (Hatch et al. 2019).

**Supplementary Table S5. Alternative maximum likelihood fits, analogous to Table 7, to assess significance of improvement in fit of various explanatory variables (to data specified by Supplementary Table S2)**^a^

| Analysis of *in utero* vs postnatal exposure | | | | | |
| --- | --- | --- | --- | --- | --- |
| Lower dose risk estimates^b^ | | | | | |
| Endpoint | ERR / Gy (95% CI) (*in utero* dose) | ERR / Gy (95% CI) (postnatal dose) | *p*-value (improvement in fit over null = no difference) | Residual heterogeneity *p*-value | *I*^2^ |
| Leukaemia | -2.70 (-11.07,5.67) | 1.31 (0.96,1.66) | 0.348 | 0.069 | 0.00 |
| Lymphoma | 229.10 (-200.94,659.14) | 0.45 (-12.83,13.74) | 0.298 | 0.981 | 0.00 |
| Brain/CNS | 70.00 (-229.37,369.37) | 6.65 (0.80,12.49) | 0.678 | <0.001 | 75.53 |
| Lung | -1.40 (-3.82,1.03) | 1.42 (-2.68,5.52) | 0.247 | 0.069 | 0.00 |
| Thyroid (including nodules) | 2.75 (1.92,3.58)^c^ | 0.00 (-0.01,0.01)^c^ | <0.001^c^ | 0.001^c^ | 0.00^c^ |
| All solid except brain/CNS, lung, thyroid | -1.31 (-2.70,0.07) | 0.00 (-0.41,0.42) | 0.074 | 0.492 | 0.00 |
| Higher dose risk estimates^d^ | | | | | |
| Endpoint | ERR / Gy (95% CI) (*in utero* dose) | ERR / Gy (95% CI) (postnatal dose) | *p*-value (improvement in fit over null = no difference) | Residual heterogeneity *p*-value | *I*^2^ |
| Leukaemia | -2.70 (-11.07,5.67) | 1.31 (0.96,1.66) | 0.348 | 0.069 | 0.00 |
| Lymphoma | 229.10 (-200.94,659.14) | 0.45 (-12.83,13.74) | 0.298 | 0.981 | 0.00 |
| Brain/CNS | 70.00 (-229.33,369.33) | 6.66 (1.20,12.13) | 0.678 | <0.001 | 82.79 |
| Lung | -1.40 (-3.82,1.03) | 0.41 (-0.23,1.06) | 0.157 | 0.483 | 0.00 |
| Thyroid (including nodules) | 1.54 (-0.15,3.22)^c^ | 0.00 (-0.01,0.01)^c^ | 0.074^c^ | <0.001^c^ | 0.00^c^ |
| All solid except brain/CNS, lung, thyroid | -1.08 (-2.75,0.59) | 0.57 (-0.37,1.51) | 0.122 | 0.065 | 44.03 |
| Analysis of low dose vs moderate+high dose studies (using L/M/H coding of maximum dose in column 3 of Tables 1-5) | | | | | |
| Lower dose risk estimates^b^ | | | | | |
| Endpoint | ERR / Gy (95% CI) (low dose) | ERR / Gy (95% CI) (moderate+high dose) | *p*-value (improvement in fit over null = no difference) | Residual heterogeneity *p*-value | *I*^2^ |
| Leukaemia | 1.31 (-4.12,6.74) | 1.30 (0.95,1.65) | 0.996 | 0.056 | 0.00 |
| Lymphoma | 9.83 (-28.54,48.19) | -0.57 (-14.73,13.58) | 0.618 | 0.869 | 0.00 |
| Brain/CNS | 6.49 (-6.62,19.60) | 6.71 (0.19,13.23) | 0.977 | <0.001 | 74.26 |
| Lung | 6.57 (-0.32,13.46) | -1.40 (-3.58,0.79) | 0.031 | 0.999 | 0.00 |
| Thyroid (including nodules) | 9.67 (3.79,15.56)^c^ | 0.00 (-0.01,0.01)^c^ | 0.001^c^ | <0.001^c^ | 0.00^c^ |
| All solid except brain/CNS, lung, thyroid | -2.39 (-8.25,3.48) | -0.09 (-0.49,0.30) | 0.444 | 0.264 | 0.00 |
| Higher dose risk estimates^d^ | | | | | |
| Endpoint | ERR / Gy (95% CI) (low dose) | ERR / Gy (95% CI) (moderate+high dose) | *p*-value (improvement in fit over null = no difference) | Residual heterogeneity *p*-value | *I*^2^ |
| Leukaemia | 1.31 (-4.12,6.74) | 1.30 (0.95,1.65) | 0.996 | 0.056 | 0.00 |
| Lymphoma | 9.83 (-28.54,48.19) | -0.57 (-14.73,13.58) | 0.618 | 0.869 | 0.00 |
| Brain/CNS | 16.63 (-1.55,34.81) | 5.67 (0.08,11.26) | 0.260 | <0.001 | 81.66 |
| Lung | NA | NA | NA | NA | NA |
| Thyroid (including nodules) | 9.94 (-2.73,22.60)^c^ | 0.00 (-0.01,0.01)^c^ | 0.124^c^ | <0.001^c^ | 0.00^c^ |
| All solid except brain/CNS, lung, thyroid | NA | NA | NA | NA | NA |
| Analysis of low dose rate vs moderate+high dose rate studies (using L/M/H coding of maximum dose rate in column 5 of Tables 1-5) | | | | | |
| Lower dose risk estimates^b^ | | | | | |
| Endpoint | ERR / Gy (95% CI) (low dose rate) | ERR / Gy (95% CI) (moderate+high dose rate) | *p*-value (improvement in fit over null = no difference) | Residual heterogeneity *p*-value | *I*^2^ |
| Leukaemia | 1.30 (0.95,1.65) | 1.03 (-3.99,6.06) | 0.917 | 0.056 | 0.00 |
| Lymphoma | 9.83 (-28.54,48.19) | -0.57 (-14.73,13.58) | 0.618 | 0.869 | 0.00 |
| Brain/CNS | 6.36 (-6.75,19.48) | 6.74 (0.23,13.25) | 0.960 | <0.001 | 74.22 |
| Lung | 6.57 (-0.32,13.46) | -1.40 (-3.58,0.79) | 0.031 | 0.999 | 0.00 |
| Thyroid (including nodules) | 0.00 (-0.01,0.01)^c^ | 7.97 (4.06,11.88)^c^ | <0.001^c^ | <0.001^c^ | 0.00^c^ |
| All solid except brain/CNS, lung, thyroid | -2.39 (-8.25,3.48) | -0.09 (-0.49,0.30) | 0.444 | 0.264 | 0.00 |
| Higher dose risk estimates^d^ | | | | | |
| Endpoint | ERR / Gy (95% CI) (low dose rate) | ERR / Gy (95% CI) (moderate+high dose rate) | *p*-value (improvement in fit over null = no difference) | Residual heterogeneity *p*-value | *I*^2^ |
| Leukaemia | 1.30 (0.95,1.65) | 1.03 (-3.99,6.06) | 0.917 | 0.056 | 0.00 |
| Lymphoma | 9.83 (-28.54,48.19) | -0.57 (-14.73,13.58) | 0.618 | 0.869 | 0.00 |
| Brain/CNS | 16.43 (-1.78,34.64) | 5.69 (0.10,11.28) | 0.271 | <0.001 | 81.65 |
| Lung | NA | NA | NA | NA | NA |
| Thyroid (including nodules) | 0.00 (-0.01,0.01)^c^ | 3.15 (1.75,4.55)^c^ | <0.001^c^ | 0.030^c^ | 0.00^c^ |
| All solid except brain/CNS, lung, thyroid | NA | NA | NA | NA | NA |

^a^using inverse-variance weighted linear model to refit thyroid nodule data <0.799 Gy of Hatch *et al* (Hatch et al. 2019), leukaemia data of Stevens *et al* (Stevens et al. 1990), and thyroid cancer data <0.284 Gy of Kopecky *et al* (Kopecky et al. 2006)

^b^using refitted thyroid nodule data <0.799 Gy of Hatch *et al* (Hatch et al. 2019), thyroid cancer data <0.284 Gy of Kopecky *et al* (Kopecky et al. 2006), Cardis *et al* (Cardis et al. 2005) thyroid cancer data using a linear model restricted to <1 Gy, Lubin *et al* (Lubin et al. 2017) data restricted to <0.1 Gy, Preston *et al* (Preston et al. 2007) brain/CNS and breast cancer data restricted to <0.1 Gy, Cahoon *et al* (Cahoon et al. 2017b) lung cancer data restricted to <0.1 Gy.

^c^indications of non-convergence for maximum likelihood fitted model.

^d^using full range thyroid nodule data of Hatch *et al* (Hatch et al. 2019), thyroid cancer data of Kopecky *et al* (Kopecky et al. 2006), Cardis *et al* (Cardis et al. 2005) thyroid cancer data using a linear model restricted to <2 Gy, Lubin *et al* (Lubin et al. 2017) data restricted to <0.2 Gy, Preston *et al* (Preston et al. 2007) brain/CNS and breast cancer data restricted to <1 Gy, Cahoon *et al* (Cahoon et al. 2017b) lung cancer data restricted to <1 Gy.

**Supplementary Table S6. Epidemiological studies of childhood cancer and natural background radiation, in which the relevant exposure is largely to gamma radiation, sometimes in conjunction with radon**

| Reference | Country (Region)^a^ | Notes | Diseases | Study Design | Agent | Cases /Controls | Mean dose (Gy) | Max dose (Gy)^b^ | Mean dose rate (nGy/h) | Max dose rate (nGy/h)^c^ |
| --- | --- | --- | --- | --- | --- | --- | --- | --- | --- | --- |
| (Sakka 1979) | Japan (Miyagi Prefecture) | Mean and maximum dose rates from Sakka (Sakka 1978), cumulative doses derived from these assumed to apply over ages 0-4 | All childhood cancers | Ecological | Gamma | 72 / NA | 0.00368 | 0.00460 (L) | 84 | 105 (L) |
| (Stjernfeldt et al. 1987) | Sweden (Östergötland) | Mean cumulative doses and dose rates in controls, maximum cumulative doses and dose rates in cases and controls | Leukaemia | Case/Control | Radon & Gamma | 15 / 15 | 0.001369 | 0.007 (L) | 43.71 | 119.8 (L) |
| (Knox et al. 1988) | Great Britain | Mean and maximum cumulative dose via Green *et al* (Green et al. 1989) | All cancers | Ecological | Gamma | 22,351 / NA | 0.0014 | 0.008 (L) | 33.9 | 82 (L) |
| (Muirhead et al. 1991) | Great Britain | Mean and maximum cumulative dose and dose rates via Wrixon *et al* (Wrixon et al. 1988) and Green *et al* (Green et al. 1989) | Leukaemia and NHL | Ecological | Radon & Gamma | 6691 / NA | 0.004 | 0.031 (L) | 96 | 270 (L) |
| (Muirhead et al. 1992) | Great Britain | Mean and maximum cumulative dose and dose rates via Wrixon *et al* (Wrixon et al. 1988) and Green *et al* (Green et al. 1989) | Leukaemia and NHL | Ecological | Radon & Gamma | 6691 / NA | 0.004 | 0.031 (L) | 96 | 270 (L) |
| (Richardson et al. 1995) | Great Britain | Mean and maximum cumulative dose and dose rates via Wrixon *et al* (Wrixon et al. 1988) and Green *et al* (Green et al. 1989) | Leukaemia | Ecological | Radon & Gamma | 6691 / NA | 0.004 | 0.031 (L) | 96 | 270 (L) |
| (Gilman and Knox 1998) | Great Britain | Mean and maximum cumulative dose and dose rates via Wrixon *et al* (Wrixon et al. 1988) and Green *et al* (Green et al. 1989) | All cancers | Ecological | Radon & Gamma | 9363 / NA | 0.005 | 0.025 (L) | 93 | 270 (L) |
| (Axelson et al. 2002) | Sweden | Mean derived from midpoint dose estimates weighted by numbers of controls | Acute lymphocytic leukaemia | Case/Control | Gamma | 312 / 1418 | 0.005303 | >0.012 (L) | NA | NA (L) |
| (United Kingdom Childhood Cancer Study Investigators 2002) | Great Britain |  | All childhood cancers | Case/Control | Gamma | 2165 / 5086 | NA | NA (L) | 96.169 | 231.24 (L) |
| (Evrard et al. 2006) | France |  | Leukaemia | Ecological | Radon & Gamma | 5330 / NA | NA | NA (L) | 142.60 | 277.21 (L) |
| (Kendall et al. 2013) | Great Britain | Mean equivalent RBM dose and range including dose from radon and gamma | All childhood cancer | Case/Control | Radon & Gamma | 27,447 / 36,793 | 0.004 | 0.031 (L) | NA | 159.7 (L) |
| (Spycher et al. 2015) | Switzerland | Gamma only | All childhood cancer | Census-based Cohort | Gamma | 1782 / NA | 0.00906 | 0.0494 (L) | 109 | 383 (L) |
| (Nikkilä et al. 2016) | Finland | Median doses, dose rates in controls | Leukaemia | Case/Control | Gamma | 1093 / 3279 | 0.0019 | >0.011 (L) | 66.4 | >140 (L) |
| (Demoury et al. 2017) | France | Mean, max dose and mean, max dose rate estimated from controls age 15 in Geocap case-control study | Acute leukaemia | Ecological | Radon & Gamma | 9056 / NA | 0.0158 | 0.0402 (L) | 98.2 | 254.7 (L) |
| (Demoury et al. 2017) | France | Mean, max dose estimated from controls age 15 | Acute leukaemia | Case/Control | Radon & Gamma | 2763 / 30,000 | 0.0158 | 0.0402 (L) | 98.2 | 254.7 (L) |
| (Spix et al. 2017) | Germany |  | Leukaemia, CNS, thyroid | Ecological | Gamma | 22,652 / NA | NA | NA (L) | 93.203 | 172 (L) |
| (Berlivet et al. 2020) | France |  | CNS | Ecological | Radon & Gamma | 5471 / NA | 0.0063 | 0.0352 (L) | 92.2 | 254.8 (L) |
| (Berlivet et al. 2021) | France | Cumulative gamma dose | Acute leukaemia | Ecological | Radon & Gamma | 6059 / NA | 0.0047 | 0.0324 (L) | 90.8 | 254.8 (L) |

^a^Where no region is shown the study covered the whole of the country.

^b^(L)=maximum dose consistent with low dose, (M)=maximum dose consistent with moderate dose but not with low dose, (H)=maximum dose consistent with high dose but not with moderate or low dose

^c^(L)=maximum dose rate consistent with low dose rate, (M)= maximum dose rate consistent with moderate dose rate but not with low dose rate, (H)= maximum dose rate consistent with high dose rate but not with moderate or low dose rate

**Supplementary Table S7. Studies of *in utero* exposure with adjustment for attained age^a^**

| Reference | Endpoint | Description | Notes | Mean / maximum attained age (years) | ERR / Gy (95% CI) | Adjusted ERR / Gy (95% CI)^a^ |
| --- | --- | --- | --- | --- | --- | --- |
| (Hagstrom et al. 1969) | Leukaemia mortality | Women administered ^59^Fe in pregnancy at Vanderbilt University Hospital, 1945-1949 and followed up to 1967 | Dose rate estimated via theoretical calculation based on physical half life of ^59^Fe, excess relative risk derived via dividing excess odds ratio by mean dose | 17.8099 / 22 | +∞ (-9.74, +∞) | +∞ (-37.44, +∞) |
|  | Lymphoma mortality |  |  |  | +∞ (-9.74, +∞) | +∞ (-37.44, +∞) |
|  | Solid tumour mortality |  |  |  | +∞ (-8.08, +∞) | +∞ (-30.79, +∞) |
| (Bithell and Stiller 1988) | All cancer mortality | Oxford Survey of Childhood Cancers, case-control pairs born 1953-1972, estimated via jointly fitted log linear model of OR with log-linear model of dose based on UNSCEAR (United Nations Scientific Committee on the Effects of Atomic Radiation (UNSCEAR) 1972) estimates | Trimester 2 | 6.37 / 15 | 20.8 (0.27, 61.8) | 20.8 (0.27, 61.8) |
|  |  |  | Trimester 3 |  | 28.8 (17.1, 43.6) | 28.8 (17.1, 43.6) |
|  |  |  | All trimesters |  | 111 (40, 397) | 111 (40, 397) |
| (Bithell 1993) | All cancer mortality | Oxford Survey of Childhood Cancers, case-control pairs born 1953-1979 | Estimated via log linear-quadratic model of OR fitted to data of Gilman *et al* (Gilman et al. 1988) by year for 1959, using dose estimate of 6.1 mGy per obstetric radiograph of Mole (Mole 1990) for that year | 6.37 /15 | 51 (28, 76) | 51 (28, 76) |
| (Delongchamp et al. 1997) | Leukaemia mortality | LSS *in utero* mortality with DS86 doses, followed up to age 47 or 1997 | Mean age derived by dividing person years follow-up by number of persons. Restricted to maternal uterine dose < 1 Gy, refitted via Poisson linear model stratified by sex, using published data. Upper profile bound uses Self and Liang (Self and Liang 1987) adjustment as MLE is on boundary | 37.81 / 47 | -1.33 (<-1.33, 87.33) | -13.73 (<-13.73, 899.59) |
|  | Solid cancer mortality |  | Mean age derived by dividing person years follow-up by number of persons. Restricted to maternal uterine dose < 1 Gy, refitted via Poisson linear model stratified by sex, using published data |  | 0.25 (-1.24, 5.35) | 2.58 (-12.57, 54.25) |
| (Johnson et al. 2008) | Leukaemia | US Radiologic Technologists offspring born 1921-1984 | Mean age derived by dividing person years follow-up by number of persons. Refitted from pubished data via Poisson linear model | 17.97 / 20 | 25.52 (-105.30, 327.70) | 99.28 (-409.66, 1274.89) |
|  | Lymphoma |  |  |  | 229.10 (-36.17, 823.90) | 891.29 (-140.72, 3205.31) |
|  | Solid cancer |  |  |  | 14.17 (-90.45, 213.20) | 54.61 (-348.62, 821.72) |
|  | All cancer |  |  |  | 56.29 (-40.26, 203.90) | 216.96 (-155.17, 785.88) |
| (Preston et al. 2008) | Solid | LSS *in utero* cohort, DS02 doses, followed to 1999 or age 55 | Mean age derived by dividing person years follow-up by number of persons. Refitted to published data maternal uterine dose < 1 Gy via Poisson linear relative risk model | 31.88 / 55 | 1.45 (-0.04, 3.88) | 11.76 (-0.31, 31.50) |
| (Bunch et al. 2009) | Leukaemia and NHL | Offspring of female members of UK National Registry for Radiation Workers | Refitted from published data via linear binomial odds model | NA / 15 | 0.00 (-1087, 41320) | 0.00 (-5548.7, 126880.9) |
|  | Cancers other than leukaemia and NHL |  |  |  | 0.00 (-5.674 x 10^7^, 5.674 x 10^7^) | 0.00 (-1.729 x 10^8^, 1.729 x 10^8^) |
| (Akleyev et al. 2016) | Solid cancer | Techa River and Mayak Worker cohorts 1950-2009 | Mean age derived by dividing person years follow-up by number of persons | 35.06 / 60 | -2.00 (-4.00, 1.00) | -18.38 (-36.75, 9.19) |
|  | Solid cancer mortality |  |  | 35.75 / 60 | -2.00 (-6.00, 1.00) | -18.85 (-56.55, 9.42) |
| (Schüz et al. 2017) | Haematological malignancy | Techa River and Mayak workers exposed/followed-up 1953-2009 (TR incidence), 1950-2009 (TR mortality), 1948-2009 (MW) | Mean age derived by dividing person years follow-up by number of persons | 35.86 / 61 | 7.70 (0.20, 25.60) | 73.99 (1.92, 245.99) |
|  | Haematological malignancy mortality |  |  | 35.39 / 61 | 1.60 (-0.90, 11.90) | 15.11 (-8.50, 112.40) |
| (Hatch et al. 2019) | Thyroid | Ukraine *in utero* ^131^I exposed cohort |  | 27 / 30 | 3.91 (-1.49, 65.66) | 25.58 (-9.75, 429.58) |
|  | Thyroid nodule |  |  |  | 1.53 (0.22, 3.59) | 10.01 (1.44, 23.49) |
|  | Thyroid nodule |  | Refitted using binomial odds model using data from paper < 0.799 Gy |  | 2.55 (0.66, 5.36) | 16.65 (4.29, 35.09) |
|  | Thyroid nodule |  | Refitted using inverse-variance reweighted least squares model using data from paper < 0.799 Gy |  | 2.75 (1.92, 3.58) | 18.01 (12.59, 23.44) |
| (Pasqual et al. 2020) | Brain/CNS | MOBI-Kids multinational case-control study of medical diagnostic radiation exposures among persons aged 10-24 y, OR of >5 mGy vs < 5 mGy | Dose lag 2 y, ERR derived by dividing ERR for > 5 mGy vs 0-5 mGy by 0.005 | NA / 24 | 70.00 (-96.00, 502.00) | 392.95 (-538.90, 2818.02)^b^ |
| (Sugiyama et al. 2021) | Solid cancer mortality | LSS *in utero* mortality with DS02R1 doses, followed up 1950-2012, maternal uterine dose | Mean age derived by dividing person years follow-up by number of persons. Refitted to published data maternal uterine dose < 1 Gy via Poisson linear relative risk model, stratified by sex | 56.11 / 67 | -0.41 (-1.34, 0.93) | -6.99 (-22.64, 15.72) |
|  | Esophageal cancer mortality |  |  |  | 1.21 (-2.23, 16.09) | 20.51 (-37.68, 272.47) |
|  | Stomach cancer mortality |  |  |  | 0.01 (-1.87, 4.28) | 0.17 (-31.70, 72.39) |
|  | Colon cancer mortality |  |  |  | -0.13 (-2.40, 7.95) | -2.28 (-40.61, 134.61) |
|  | Rectal cancer mortality |  |  |  | 1.40 (-2.20, 17.12) | 23.71 (-37.26, 289.91) |
|  | Liver cancer mortality |  |  |  | -2.10 (-2.76, 0.65) | -35.63 (-46.72, 10.98) |
|  | Pancreas cancer mortality |  |  |  | 1.40 (-2.20, 16.78) | 23.62 (-37.24, 284.16) |
|  | Lung cancer mortality |  |  |  | -1.40 (-2.58, 2.26) | -23.64 (-43.66, 38.34) |
|  | Lymphohaemopoietic mortality |  |  |  | 4.45 (-1.90, 60.14) | 76.87 (-32.78, 1038.57) |
|  | Leukaemia mortality |  |  |  | -2.80 (-2.84, 14.22) | -48.30 (-49.10, 245.57) |

^a^using [mean attained age]^-1.3^ to adjust all solid cancer (and thyroid nodule) ERR to mean attained age of 6.37 years in study of Bithell and Stiller (Bithell and Stiller 1988) and Bithell (Bithell 1993), and [mean attained age]^-1.309^ to adjust for all haemopoietic malignancies, derived via fit of stratified linear-quadratic ERR model to all radiogenic leukaemias (acute lymphocytic leukaemia, acute myeloid leukaemia, chronic myeloid leukaemia) exposed under the age of 20, with adjustment for ln[attained age], using strata of city, sex, age at exposure, age and calendar time in data of Hsu *et al* (Hsu et al. 2013), omitting persons not in either city (Hiroshima, Nagasaki) at the time of the bombings, or those with shielded kerma dose ≥ 4 Gy.

^b^using maximum attained age rather than mean attained age to adjust.

**Supplementary Table S8. Egger test (Egger et al. 1997) for selection bias, and magnitude of correction suggested by trim-and-fill method of Duval and Tweedie (Duval and Tweedie 2000), employing lower and higher doses sets of risks as in Tables 6, 7**

| Endpoint | Egger test for selection bias | ERR (95% CI) (raw maximum likelihood estimate) | Bias corrected ERR (95% CI) (Duval & Tweedie trim-and-fill corrected maximum-likelihood estimate) |
| --- | --- | --- | --- |
| Lower dose risk estimates | | | |
| All post-natal | <0.001 | 0.00 (-0.01,0.01) | 1.59 (-1.02,4.19) |
| All *in utero* | 0.322 | -0.29 (-1.84,1.26) | -0.31 (-1.85,1.24) |
| Leukaemia | 0.023 | 1.30 (0.95,1.65) | 1.29 (0.94,1.64) |
| Lymphoma | 0.189 | 1.42 (-11.74,14.59) | 0.14 (-12.75,13.02) |
| Brain/CNS | 0.094 | 6.67 (0.83,12.52) | 4.97 (-0.90,10.84) |
| Lung | 0.102 | -0.67 (-2.75,1.42) | -0.67 (-2.75,1.42) |
| Thyroid (including nodules) | <0.001 | 0.00 (-0.01,0.01) | 0.00 (-0.01,0.01) |
| Solid other than lung, brain/CNS, thyroid | 0.984 | -0.10 (-0.50,0.29) | -0.10 (-0.50,0.29) |
| Solid other than thyroid | <0.001 | 1.89 (0.10,3.69) | 1.47 (-0.31,3.26) |
| Higher dose risk estimates | | | |
| All post-natal | <0.001 | 3.34 (1.64,5.05) | 1.28 (-1.26,3.83) |
| All *in utero* | 0.377 | -0.39 (-1.78,1.00) | -0.40 (-1.79,0.98) |
| Leukaemia | 0.023 | 1.30 (0.95,1.65) | 1.29 (0.94,1.64) |
| Lymphoma | 0.189 | 1.42 (-11.74,14.59) | 0.14 (-12.75,13.02) |
| Brain/CNS | 0.044 | 6.69 (1.22,12.15) | 5.18 (-0.27,10.63) |
| Lung | 0.154 | 0.29 (-0.33,0.92) | 0.44 (-0.16,1.04) |
| Thyroid (including nodules) | <0.001 | 0.00 (-0.01,0.01) | 0.40 (-2.60,3.40) |
| Solid other than lung, brain/CNS, thyroid | 0.474 | 0.22 (-0.82,1.26) | -0.33 (-1.44,0.77) |
| Solid other than thyroid | <0.001 | 1.51 (0.11,2.91) | 1.23 (-0.14,2.60) |

**Supplementary Table S9. PECO statement^a^**

| PECO element | Evidence stream | Articles or features included | Articles or features excluded |
| --- | --- | --- | --- |
| Population | Human | Any population  Exposure *in utero* or in childhood (age at exposure 20 y or less)  Maximum cumulative organ dose ≤ 1 Gy OR maximum organ dose rate ≤ 0.1 Gy / hour  Study designs   1. Cohort 2. Case-cohort 3. Case-control 4. Nested case-control 5. Cross sectional | Predominant exposure is from radon or other high LET radiation  Cumulative maximum organ dose > 1 Gy AND maximum organ dose rate > 0.1 Gy / hour  Case series  Ecologic studies  Mechanistic studies  Information on risks in relation to exposure age ≤20 y not given |
|
|
|
|
|
|
| Exposure | Human | Ionizing radiation exposure   1. Gamma rays 2. X-rays 3. Beta rays 4. Other low LET | Semiquantitative or qualitative dose response |
|
|
| Comparator | Human | A comparison population, exposed to lower level of radiation exposure, or presumed largely unexposed (e.g. national population) | No comparison group |
|
|
|
|
| Outcome | Human | Cancer endpoints (deaths, incidence)  Benign tumours (deaths, incidence) | Cancer endpoints not described  Benign tumour endpoints not described |
| General considerations |  | Reports primary source  Full text available | Reports secondary source (e.g. review articles)  Editorials  Only abstracts  Correspondence |
|
|
|
|
|
|
|

^a^PECO = Population, Exposure, Comparator and Outcome

**Supplementary Figure S1. Funnel plots of excess relative risks / Gy, all endpoints and studies (*in utero* and post-natal exposure) (using all data as in Tables 1-5, employing higher dose sets of risks as in Tables 6, 7).**

| Postnatal exposure | *In utero* exposure |
| --- | --- |
| 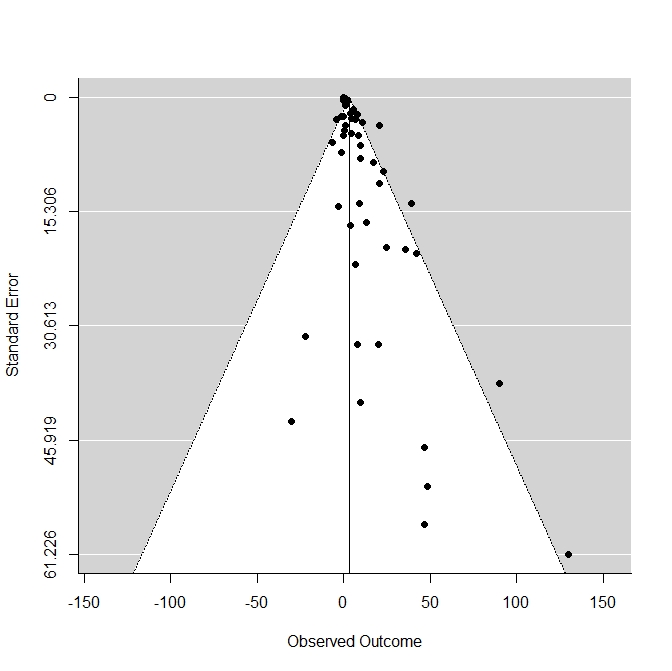 | 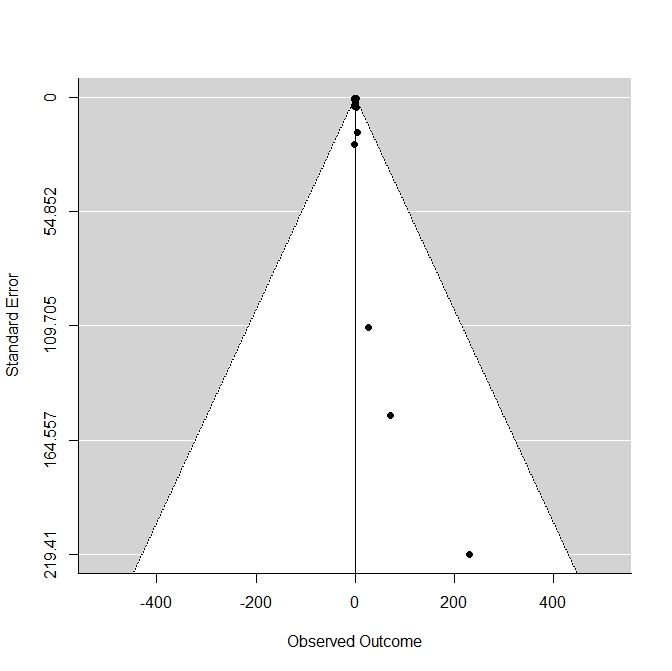 |
